# Gut microbiota‐derived butyric acid regulates calcific aortic valve disease pathogenesis by modulating GAPDH lactylation and butyrylation

**DOI:** 10.1002/imt2.70048

**Published:** 2025-05-19

**Authors:** Chunli Wang, Zongtao Liu, Tingwen Zhou, Jiaqin Wu, Fan Feng, Shunshun Wang, Qingjia Chi, Yongqiang Sha, Shuai Zha, Songren Shu, Linghang Qu, Qianqian Du, Huiming Yu, Li Yang, Anna Malashicheva, Nianguo Dong, Fei Xie, Guixue Wang, Kang Xu

**Affiliations:** ^1^ Hubei Shizhen Laboratory Wuhan China; ^2^ School of Laboratory Medicine Hubei University of Chinese Medicine Wuhan China; ^3^ Department of Cardiovascular Surgery, Union Hospital, Tongji Medical College Huazhong University of Science and Technology Wuhan China; ^4^ School of Pharmacy Hubei University of Chinese Medicine Wuhan China; ^5^ College of Electrical Engineering and Automation Anhui University Hefei China; ^6^ Center for Precision Medicine, School of Medicine and School of Biomedical Sciences Huaqiao University Xiamen China; ^7^ Fuwai Hospital, National Center for Cardiovascular Diseases Chinese Academy of Medical Sciences and Peking Union Medical College Beijing China; ^8^ Ministry of Education Key Laboratory for Biorheological Science and Technology, National Local Joint Engineering Lab for Vascular Implants, College of Bioengineering Chongqing University Chongqing China; ^9^ Institute of Cytology Russian Academy of Science Petersburg Russia; ^10^ Department of Cardiovascular Surgery The First Affiliated Hospital of Zhengzhou University Zhengzhou China; ^11^ JinFeng Laboratory Chongqing China

**Keywords:** butyric acid, butyrylation, calcific aortic valve calcification, glycolysis, lactylation

## Abstract

The involvement of gut microbiota in calcific aortic valve disease (CAVD) pathogenesis remains underexplored. Here, we provide evidence for a strong association between the gut microbiota and CAVD development. ApoE^−/−^ mice were stratified into easy‐ and difficult‐ to calcify groups using neural network and cluster analyses, and subsequent faecal transplantation and dirty cage sharing experiments demonstrated that the microbiota from difficult‐to‐calcify mice significantly ameliorated CAVD. 16S rRNA sequencing revealed that reduced abundance of *Faecalibacterium prausnitzii* (*F. prausnitzii*) was significantly associated with increased calcification severity. Association analysis identified *F. prausnitzii*‐derived butyric acid as a key anti‐calcific metabolite. These findings were validated in a clinical cohort (25 CAVD patients vs. 25 controls), where serum butyric acid levels inversely correlated with disease severity. Functional experiments showed that butyric acid effectively hindered osteogenic differentiation in human aortic valve interstitial cells (hVICs) and attenuated CAVD progression in mice. Isotope labeling and ^13^C flux analyses confirmed that butyric acid produced in the intestine can reach heart tissue, where it reshapes glycolysis by specifically modifying GAPDH. Mechanistically, butyric acid‐induced butyrylation (Kbu) at lysine 263 of GAPDH competitively inhibited lactylation (Kla) at the same site, thereby counteracting glycolysis‐driven calcification. These findings uncover a novel mechanism through which *F. prausnitzii* and its metabolite butyric acid contribute to the preservation of valve function in CAVD, highlighting the gut microbiota‐metabolite‐glycolysis axis as a promising therapeutic target.

## Highlights

- The inseparable relationship between the gut microbiota and calcific aortic valve disease development.
- *Faecalibacterium prausnitzii* (*F. prausnitzii*)‐derived butyric acid (BA) played an important role in anti‐calcification functions.
- BA‐derived butyrylation (Kbu) blocked lactylation (Kla) at the same site by occupying the GAPDH 263 lysine.
- The gut microbial‐metabolite‐epigenetic modification pathway represents a promising therapeutic target for calcific aortic valve disease (CAVD).

## Graphical abstract

Multi‐omics sequencing and correlation analysis identified the beneficial role of *Faecalibacterium prausnitzii* (*F. prausnitzii*)‐derived butyric acid (BA) as a key metabolite in the restoration of valve function in calcific aortic valve disease (CAVD). The therapeutic efficacy of BA in attenuating CAVD progression was confirmed in vitro, ex vivo, and in vivo. Subsequent mechanistic investigations revealed that BA reshape glycolysis through site‐specific inhibition of lactylation at the Lys‐263 residue of GAPDH, which is mediated by competitive inhibition of butyrylation at the same site.

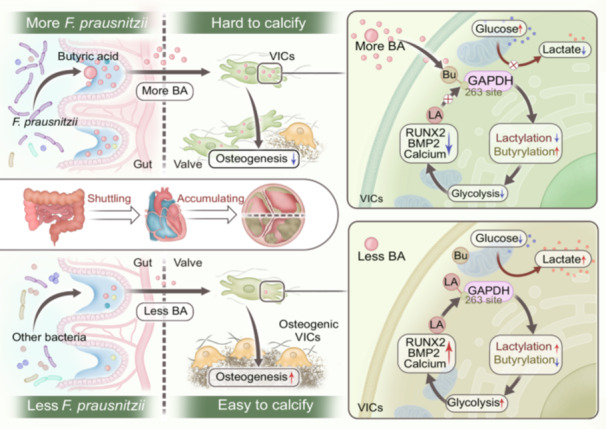

## INTRODUCTION

Calcific aortic valve disease (CAVD) is defined as a complex and chronic condition that comprises a continuum of pathological changes ranging from microscopic changes to a buildup of the extracellular matrix, aortic thickening, stenosis, and arrhythmia, culminating in heart failure and premature death [1, 2]. Despite the significant clinical burden, no pharmacotherapy has yet demonstrated efficacy in halting the progression of CAVD, with invasive and costly aortic valve replacement remaining the sole treatment option currently available. This substantial challenge in curing this disease is largely because of our still‐limited understanding of valvular homeostasis and regulatory mechanisms that drive disease initiation and progression [3, 4, 5]. Drug development is further hampered by the diverse spectrum of risk factors and the inherent intricacy of the valvular microenvironment: a heterogeneous cell subpopulation and complex anatomical structure collectively contribute to this currently intractable problem [6, 7, 8]. Hence, the prerequisite for a promising and rapidly evolving anti‐valve calcification tactic is to fully define disease pathogenesis.

Large‐scale studies have indicated that genetic variations contribute to only a minor portion of disease susceptibility [9]. Current well‐documented evidence has demonstrated that the gut microbiota has emerged as an influential factor affecting human health and disease [10, 11]. Alterations in the gut microbial community composition and structure are associated with numerous disease states, and cardiovascular disease (CVD) is not an exception to this trend. Numerous researchers have identified associations between atherosclerosis phenotypes and alterations in the relative abundance of specific microbial taxa, as well as changes in gut bacterial richness or diversity [12, 13]. Indeed, the development of drugs that target diseases through modulation of the gut microbiota is becoming increasingly prominent. Nonetheless, the relationship between the gut microbiota and CAVD progression has not been investigated; therefore, exploring the role of the gut microbiota in mediating valvular fibrosis and calcification has opened avenues for therapeutic interventions that may improve the prevention and treatment of now ubiquitous CAVD.

While bacterial protein degradation products have traditionally been viewed as detrimental to the host, recent evidences indicate that metabolites produced by the gut microbiota play crucial roles in maintaining intestinal homeostasis and metabolic health [14]. In addition to serving as an energy source for gut epithelial cells, these metabolites are absorbed into the portal circulation, where they engage in a range of physiological and pathological processes within the host [15]. They are instrumental in regulating lipid metabolism, glucose homeostasis, and inflammation. Metabolic pathways link the gut microbiota to the altered phenotypes of cells during disease. Indeed, the gut microbiota plays either protective or harmful roles in CVD development by generating metabolites, which are essential components involved in the crosstalk between microorganisms and the host [16]. However, no studies have investigated whether gut microbiota‐derived metabolites play a role in the occurrence and development of CAVD.

Here, continuously exposing mice to dirty cages was designed to first determine if the gut microbiota was pathologically linked to CAVD and then to evaluate whether faecal transplantation transformed disease states. Faecal samples were collected from difficultly calcified and easy calcified mice, and we sequenced stool samples, which were representative of the gut microbiota, and blood samples, which were representative of metabolites. Integrative association analyses revealed reduced *Faecalibacterium prausnitzii g Faecalibacterium* (*F. prausnitzii*) and butyric acid (BA) levels in difficult calcification model mice. The oral administration of *F. prausnitzii* and butyric acid in a mouse model of CAVD demonstrated their beneficial effects on valve function. We demonstrated that the mechanism by which *F. prausnitzii* prevents CAVD progression involves its metabolite butyric acid. Isotope labeling experiments (ILEs) and ^13^C flux analysis indicated that labeled butyric acid in the intestine is shuttled to heart tissue, where butyric acid specifically represses Kla at the lysine 263 site of GAPDH, accompanied by an increase in Kbu at the GAPDH K263 site. Mechanistically, the competitive binding of Kbu and Kla at GAPDH results in a decrease in lactylation, thereby retarding the osteogenic differentiation mediated by abnormal glycolysis.

## RESULTS

### Aortic valve calcification is modulated by fecal microbiota and cage environment

**FIGURE 1:**
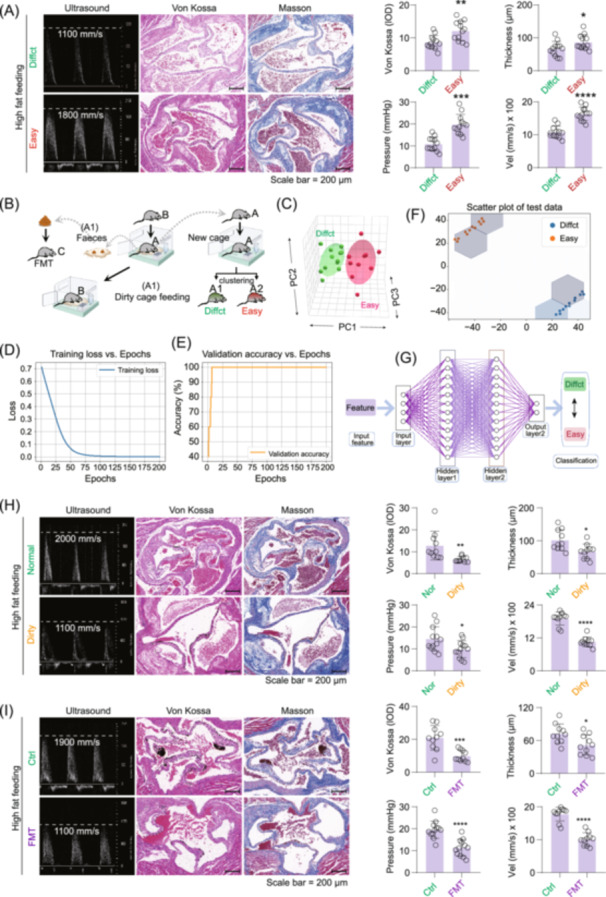
Aortic valve calcification is closely related to the gut microbiota. (A) Comparison of the degree of heart valve calcification in the difficult calcification and easy calcification groups of ApoE^−/−^ mice after a high‐fat diet. (B) Flowchart of the dirty cage experiment and faecal microbiota transplantation. (C) The principal component analysis (PCA) and orthogonal partial least squares discriminant analysis (OPLS‐DA) cluster analysis were used to group high‐fat diet‐fed mice (difficult calcification and easy calcification mice) on the basis of Von Kossa staining (IOD) of mouse aortic valves, valve thickness, flow velocity (mm/s) and transvalvular pressure difference (mmHg). (D–G) Neural network analysis was used to validate the results of PCA and OPLS‐DA cluster analyses. (H) Squirrel cages from the difficult calcification group (B group in Figure B) were utilized to feed other high‐fat‐fed ApoE^−/−^ mice (Dirty group). ApoE^−/−^ mice fed in clean cages were used as the control group (Normal group). (I) Faecal microbiota transplantation (FMT) was performed on other high‐fat‐fed ApoE^−/−^ mice (C group in Figure B), the faeces collected from the difficult calcification group (A1 group in Figure B). The non‐FMT mice were used as the control group. One‐way analysis of variance (ANOVA) was employed for multiple comparisons, whereas *t* tests were used for two‐group comparisons. *p < 0.05, **p < 0.01, ***p < 0.001 indicate significant differences compared with the control group, *n* = 12. Scale bar: 200 μm.

Significant differences in calcification levels were observed among mice housed in individual cages and fed a high‐fat (HF) diet, based on transvalvular differential pressure, flow velocity, calcium deposition, and valve thickness measurements (Figure 1A,B). Clustering analysis based on these parameters revealed two distinct phenotypes: one group of mice (*n* = 12) with low susceptibility to calcification (Diffct), and another group (*n* = 12) with high susceptibility (Easy), as further supported by principal component analysis (PCA) and orthogonal partial least squares discriminant analysis (OPLS‐DA) (Figures 1C, S1). Neural network validation of the classification achieved 100% training accuracy after several iteration (Figures 1D–F, S1C). A classification plot generated through dimensionality reduction confirmed clear separation between the two groups, verify the robustness of the grouping (Figure 1F,G).

To explore environmental influences on calcification, two additional groups of mice were studied. One was continuously housed in standard cages (Nor), while the other was housed in “dirty” cages previously used by mice from the Diffct group (Dirty). Mice in the Dirty group exhibited significantly lower calcium deposition, valve thickness, pressure gradients, and flow velocity compared with those in the Nor group (Figure 1H). Notably, faecal microbiota transplantation (FMT) reproduced the protective effects observed with dirty cage housing, ameliorating calcification levels in HF diet‐fed mice (Figure 1I). The experimental workflows for the dirty cage and FMT interventions were illustrated in Figure 1B.

### *Faecalibacterium prausnitzii* is the key strain inhibiting valve calcification in the faeces of ApoE^−/−^ mice

**FIGURE 2:**
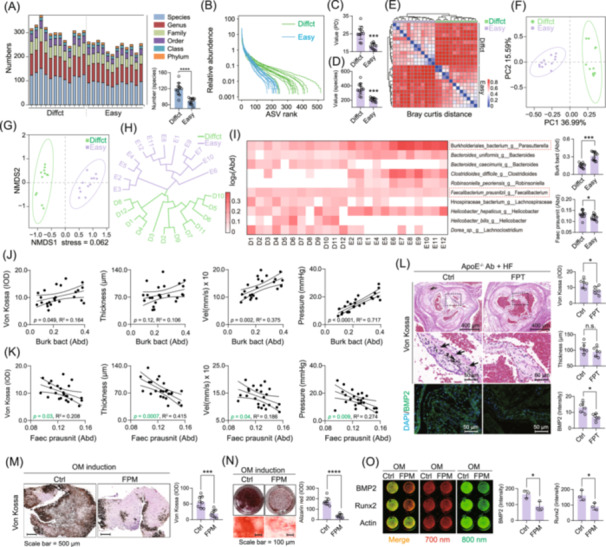
Gut microbial sequencing identified *Faecalibacterium prausnitzii* (*F. prausnitzii*) as the key species in faeces that inhibits valve calcification. (A) Community structure analysis with statistical results of the number of species annotated to the phylum order family species level in each sample, in which the microbial species in the faeces of easy calcification (Easy) mice were significantly lower than those of difficult calcification (Diffct) mice. (B) Rank abundance (Abd) analysis showing differences in the abundance and homogeneity of the species contained in faeces samples of difficult calcification versus easy calcification mice. (C and D) Sample alpha diversity analysis. (E) Distance matrix after beta diversity analysis. (F) Taxonomic analysis via PCA. (G) Taxonomic analysis via NMDS. (H) Hierarchical clustering tree between samples. (I) Top 10 heatmaps of differential species abundance, *Burkholderiales_bacterium* (*Burk bact*) and *Faecalibacterium prausnitzii* (*F. prausnitzii*) detectable in the whole samples. (J) Multimetric correlation analysis between *Burk bact* strain abundance and valve calcification. (K) *F. prausnitzii* strain abundance and valve calcification multicriteria correlation analysis. (L) *F. prausnitzii* monoculture transplantation of ApoE^−/−^ mice and high‐fat valve calcification modeling to assess the inhibitory effect of monoculture transplantation on valve calcification. (M) Human valve tissue ex vivo samples under osteogenic medium (OM) induction were treated with the medium supernatant of *F. prausnitzii* (FPM), and the degree of calcification was evaluated by Von Kossa staining, scale bar: 500 μm. (N) FPM was used to treat human aortic valve interstitial cells (hVICs) cells under OM induction, and alizarin red staining was used to evaluate the degree of calcification, scale bar: 100 μm. (O) FPM was used to treat hVICs cells under OM induction, and in‐cell WB was used to evaluate the expression of BMP2 and Runx2 calcification markers in hVICs cells. One‐way ANOVA was employed for multiple comparisons, whereas *t* tests were used for two‐group comparisons. *p < 0.05; **p < 0.01; ***p < 0.001 indicate significant differences, *n* = 12.

16S rRNA sequencing revealed that microbial diversity was higher in the faeces of resistant to calcification (Figures 2A,B, S2A, B). Both alpha and beta diversity analyses confirmed distinct gut microbiota compositions between the calcification‐resistant (Diffct) and susceptible (Easy) groups (Figures 2C–H, S2C–E). Further analyses comparing the top 10 strains with significant differences revealed that only two species, *Burkholderiales_bacterium* g *Parasutterella* and *F. prausnitzii*, were consistently detected across all samples (Figure 2I). *Burkholderiales_bacterium* was significantly enriched in the faeces of mice susceptible to calcification and showed a positive correlation with calcification severity, suggesting a pro‐calcification role (Figure 2I,J). In contrast, the abundance of *F. prausnitzii* was significantly lower in these mice and showed a strong negative correlation with multiple calcification factors, including Von Kossa staining, valve thickness, transvalvular pressure gradient and flow velocity (*p* < 0.05) (Figure 2I,K).

To further investigate the anti‐calcification potential of *F. prausnitzii*, the bacterium was cultured and administrated as a single strain to ApoE^−/−^ mice. Von Kossa and immunofluorescence staining demonstrated a significant reduction in both aortic valve calcification and BMP2 expression in mice treated with *F. prausnitzii* compared to those on a HF diet alone (Figure 2L). Serum collected from *F. prausnitzii*‐treated ApoE^−/−^ mice was used to treat human valve interstitial cells (hVICs) under osteogenic differentiation. The addition of 10% serum significantly inhibited hVIC osteogenic differentiation induced by osteogenic medium (OM) (Figure S3A). The *F. prausnitzii* culture supernatant was subsequently tested at various dilutions, and a 10% concentration was identified as optimal (Figure S3B–F). Treatment with 10% *F. prausnitzii* supernatant significantly reduced calcification in human valve tissue cultured *ex vivo* (Figure 2M) and suppressed calcification associated with osteogenic differentiation and expression of calcification markers (BMP2, Runx2, OPN, OCN) in hVICs (Figures 2N–O, S3G–K). In addition, both the serum and supernatant exhibited inhibitory effects on aortic valve calcification in vivo (Figure S3L–M). These results suggest that *F. prausnitzii* exerts a protective, anti‐calcific effect and may serve as a probiotic for the prevention of valvular calcification.

### Butyric acid plays a key role in CAVD associated with *F. prausnitzii*

**FIGURE 3:**
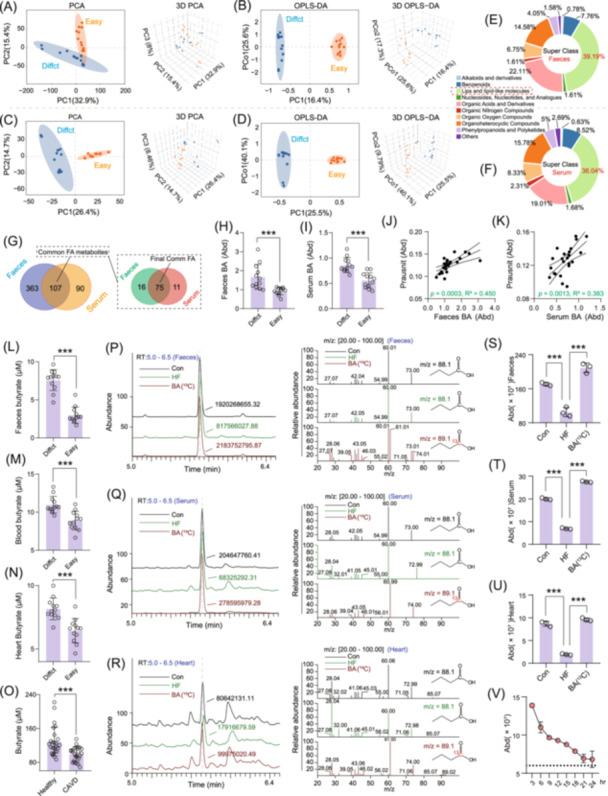
Metabolomic analysis of faeces and serum identified butyric acid as a key mediator of anticalcification in *F. prausnitzii*. (A–D) Cluster analysis of the metabolites PCA and OPLS‐DA in the faeces and serum of easy‐calcification and difficult‐calcification ApoE^−/−^ mice (*n* = 24, 12 vs. 12). (E, F) Compositional analysis of the metabolites in the faeces (*n* = 24) and serum (*n* = 24). (G) 107 common differentially abundant metabolites were identified between the serum and faeces, of which 75 common differentially abundant metabolites were detected in all the samples. (H, I) Differences in the abundance of butyric acid in the faeces and serum of the mice. (J, K) Correlation analysis of the faecal and serum abundance of butyric acid with the abundance of *F. prausnitzii*. (L–N) Determination of butyric acid in the faeces, serum and heart of the easy calcification and difficult calcification ApoE^−/−^ mice. (O) Determination of butyric acid in the serum of healthy and CAVD patients (*n* = 50). (P–R) Total ion flow and MS identification of the ^13^C‐labeled butyric acid detected in the faeces, serum and heart of the mice after oral gavage (BA: ^13^C‐labeled butyric acid oral administration group; HF: fed a high‐fat diet; control: nontreatment). (S–U) Abundance of isotope ^13^C‐labeled butyric acid detected in faeces, serum and heart. (V) Relative abundance of isotope ^13^C‐labeled butyric acid in the heart was assayed within 24 h of gavage. One‐way ANOVA was employed for multiple comparisons, whereas *t* tests were used for two‐group comparisons. ***p < 0.001, significant difference compared with the control group.

To investigate the molecular mechanisms underlying the association between *F. prausnitzii* and CAVD, we performed metabolomic analyses of faecal and serum samples from ApoE^−/−^ mice with either high (Easy, *n* = 12) and low (Diffct, *n* = 12) susceptibility to valve calcification. PCA and OPLS‐DA analyses revealed distinct clustering between the two groups, indicating significant metabolic differences in both faeces and serum (Figures 3A–D, S4). Furthermore, metabolite profiling showed that differentially abundant compounds were predominantly enriched in the lipids and lipid‐like category (Figure 3E,F). A total of 107 overlapping differentially abundant metabolites identified in both faeces and serum are lipids and lipid‐like metabolites, of which 75 were consistently detected across all samples (Figure 3G). Among these, butyric acid emerged as the most strongly correlated metabolite with *F. prausnitzii* abundance. Its levels were significantly higher in the calcification‐resistant group (Diffct) compared to the other group (Easy) and positively correlated with *F. prausnitzii* abundance in both faeces and serum (Figures 3H–K, S5).

Further analyses confirmed elevated butyric acid levels in the faeces, serum and heart tissue of calcification‐resistant mice (Figure 3L–N). Clinically, serum butyric acid levels were also assessed in human subjects (*n* = 50; 25 healthy vs. 25 CAVD patients) (Table S1). The CAVD group exhibited significantly lower butyric acid levels than the healthy controls (Figure 3O). Previous studies have established that *F Prausnitzii* is a major butyric acid‐producing bacterium in the intestine [17, 18, 19], supporting the conclusion that its protective role in CAVD may be mediated by butyric acid.

To assess the systemic distribution and functional role of butyric acid, we orally administrated ^13^C‐labeled butyric acid to ApoE^−/−^ mice and measured its levels in the faeces, serum and various organs. In HF diet‐fed mice, endogenous butyric acid levels were significantly reduced compared to controls. However, oral administration of ^13^C‐labeled butyric acid markedly increased its concentration in multiple organs, including the heart, liver, spleen, lung and kidney (Figures 3P–U, S6). Kinetic analysis showed that butyric acid levels in cardiac tissues peaked at 3 h post‐administration and gradually declined, stabilizing after 18 h (Figures 3V, S7). These findings confirm that butyric acid can cross the intestinal barrier, enter the bloodstream, and accumulate in cardiac tissues, where it may exert its protective effects against CAVD.

### Butyric acid ameliorates aortic valve calcification ex vivo and in vivo

**FIGURE 4:**
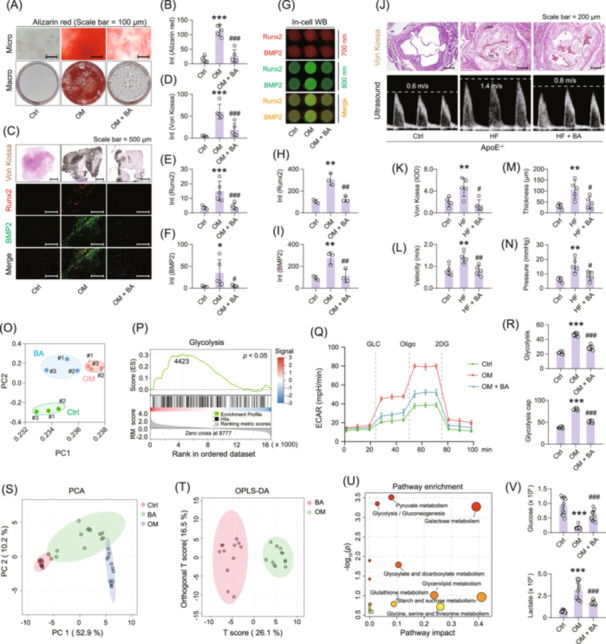
Butyric acid inhibited heart valve calcification through regulating glycolytic metabolism in hVICs. (A, B) Effects of butyric acid on calcium deposition in osteogenic medium (OM)‐induced calcific hVICs detected by alizarin red staining, scale bar: 100 μm. (C–F) Evaluation of calcification level in butyric acid‐treated human valve tissues ex vivo, including Von Kossa staining, and immunofluorescence staining of Runx2 and BMP2, scale bar: 500 μm, *n* = 5. (G–I) In‐cell western blot analysis of Runx2 and BMP2 in butyric acid‐treated hVICs under OM‐induced calcification, *n* = 3. (J–N) Evaluation of calcification level in butyric acid‐treated ApoE^−/−^ mice after high‐fat‐feeding‐induced heart valve calcification, scale bar: 200 μm, *n* = 5. (O) PCA clustering analysis of butyric acid‐treated hVICs under OM‐induced calcification utilizing RNA‐seq, *n* = 3. (P) GSEA indicated that glycolysis metabolism was significantly different in hVICs under OM‐induced calcification between butyric acid‐treated and nonbutyric acid‐treated groups. (Q, R) Extracellular acidification rates (ECAR) and glycolysis levels in hVICs of the control, OM and OM + BA groups, *n* = 3. (S, T) PCA and OPLS‐DA clusting analysis based on cellular metabolomics detection in hVICs of the control, OM and BA + BA groups. (U) KEGG analysis on different metabolites in hVICs of the control, OM and OM + BA groups. (V) Abundance of glucose and lactate in butyric acid‐treated hVICs under OM‐induced calcification. One‐way ANOVA was employed for multiple comparisons, whereas *t* tests were used for two‐group comparisons. *p < 0.05, **p < 0.01, ***p < 0.001 indicate significant differences compared with the control group; ^#^ *p* < 0.05, ^##^ *p* < 0.01, ^###^ *p* < 0.001 indicate significant differences compared with the OM or HF group.

The protective effects of butyric acid on aortic valve calcification were further evaluated *ex vivo* and in vivo. Cytotoxicity assays confirmed that butyric acid at concentrations between 400 and 800 μM had no significant toxic effects on hVICs (Figure S8A). Western blot analysis showed that butyric acid at concentrations ranging from 100 to 700 μM significantly inhibited the expression of Runx2 and BMP2 in hVICs (Figure S8B, C), with 500 μM utilized in subsequent experiments. Alizarin red staining revealed a marked calcium deposition in OM‐treated hVICs, which was substantially reduced by butyric acid treatment (Figure 4A,B). In both OM‐treated hVICs and ex vivo cultured human aortic valve tissues, butyric acid dose‐dependently suppressed the expression of Runx2 and BMP2 (Figures 4C–F, S8B–E). In‐cell western assays further confirmed that butyric acid inhibited OM‐induced expression of these calcification markers in hVICs (Figure 4G–I).

Transcriptome analysis indicated that OM treatment significantly altered the expression of 1395 genes in hVICs, while butyric acid effectively reversed the expression of 229 common genes (Figure S9A–C). These genes were primarily enriched in pathways related to metabolic regulation and cardiovascular disease (Figure S9D–G). In vivo, oral administration of butyric acid significantly inhibited aortic valve calcification in ApoE^−/−^ mice, as shown by improvement of transvalvular pressure gradients, flow velocity, calcium deposition and valve thickness (Figure 4J–N).

Gene set enrichment analysis revealed that butyric acid significantly altered glycolytic activity in the OM‐treated hVICs (Figure 4O,P). Measurements of extracellular acidification rates (ECAR) indicated that OM increased glycolytic influx, as evidenced by elevated glucose, oligomycin and 2‐deoxy‐glucose (2‐DG). Butyric acid attenuated these effects, indicating a normalization of glucose metabolism (Figure 4Q,R). Metabolomic profiling further supported these findings. OM treatment led to reduced glucose levels and increased lactate production in hVICs, consistent with enhanced glycolysis. Butyric acid treatment restored glucose levels and reduced lactate accumulation (Figure 4S–V).

Taken together, butyric acid effectively reduces aortic valve calcification both in vivo and ex vivo by downregulating osteogenic signaling pathways, reversing gene expression changes associated with calcification, and normalizing aberrant glycolytic metabolism in hVICs.

### Butyric acid inhibits aortic valve calcification by competitively blocking GAPDH lactylation

**FIGURE 5:**
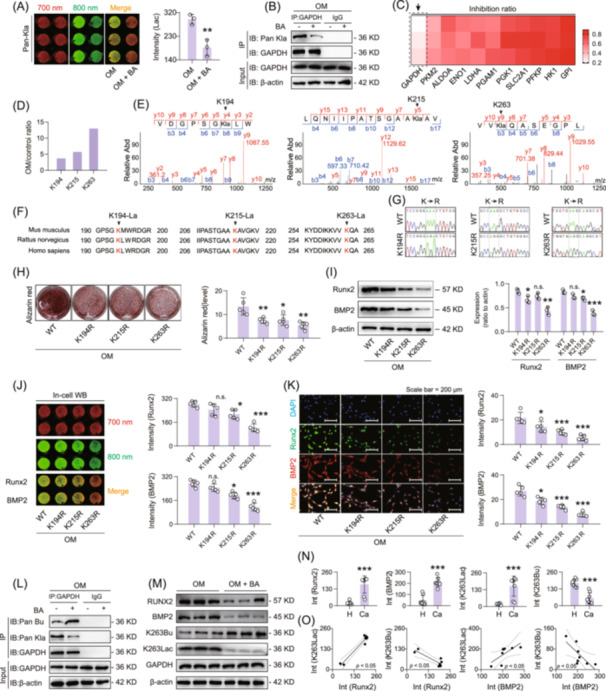
Butyric acid exerts antivalvular calcification effects by competitively inhibiting GAPDH lactylation. (A) In‐cell WB assay of global intracellular lactylation levels after treatment of osteogenic medium (OM)‐induced hVICs with butyric acid, *n* = 3. (B) The lactylation of GAPDH was most significantly inhibited by butyric acid, *n* = 3. (C) Sequencing analysis of lactylation levels of the glycolytic enzymes by butyric acid under OM induction, *n* = 3. (D, E) Histological analysis of hVICs subjected to OM induction revealed three significant lactylation sites in GAPDH. (F) Homology analysis of the three lactylated sites in different species. (G) Sequencing analysis of mutant plasmids of the three lactylated sites. (H) Alizarin red staining was used to analyze the role of three lactylated site mutations in regulating valve calcification, *n* = 5. Analysis of the role of three lactylated site mutations in regulating valve calcification via western blot analysis, *n* = 3 (I), in‐cell WB (J) and immunofluorescence staining (K) for Runx2 and BMP2 protein expression, scale bar: 200 μm, *n* = 5. (L) Immunoprecipitation analysis of butyric acid treatment affecting the butyrylation and lactylation level of GAPDH, *n* = 3. (M) Changes in the expression of GAPDH K263Bu/K263Lac and the calcification marker Runx2/BMP2 after butyric acid treatment. (N) Expression analysis of K263 butyrylation and lactylation with the calcification marker Runx2/BMP2 in hVICs collected from eight calcified patients versus eight healthy patients. (O) Correlation analysis of K263 butyrylation and K263 lactylation co‐expression with the calcification marker Runx2/BMP2, respectively. One‐way ANOVA was employed for multiple comparisons, whereas *t* tests were used for two‐group comparisons. *p < 0.05, **p < 0.01, ***p < 0.001 indicate significant differences compared with the control group.

To explore the mechanistic role of butyric acid in regulating valve calcification, we examined its effect on protein lactylation. In‐cell western blot analysis using a pan‐lysine lactylation (Kla) antibody revealed that butyric acid significantly reduced global protein lactylation in hVICs under osteogenic stimulation (Figure 5A). Lactylome profiling revealed that lactylation of several glycolytic enzymes was prominently involved in the process of valve calcification (Table S2). Among these, glyceraldehyde‐3‐phosphate dehydrogenase (GAPDH) emerged as a key target. Butyric acid inhibited lactylation levels of multiple glycolytic enzymes in OM‐induced hVICs (Figure S10), with the most substantial effect observed on GAPDH (Figure 5B,C). Mass spectrometry (MS) analysis further identified three major lactylation sites on GAPDH, K194, K215 and K263 (Figure 5D,E). These lysine residues are evolutionarily conserved, indicating potential structural and functional importance (Figure 5F).

To functionally validate these sites, we generated GAPDH mutants (K194R, K215R, K263R) to mimic a delactylated state (Figure 5G). Alizarin red staining showed that all three mutations significantly reduced calcium deposit in OM‐induced hVICs, with K263R having the most pronounced effect (Figure 5H). Consistent with this, both western blot and immunofluorescence analyses confirmed that the expression of Runx2 and BMP2 was significantly suppressed in cells expressing GAPDH K263R (Figure 5I–K). Immunoprecipitation experiments revealed that exogenous butyric acid significantly inhibited GAPDH lactylation while simultaneously increasing its butyrylation in OM‐induced hVICs (Figure 5L). To explore this further, we developed site‐specific antibodies against lactylated and butyrylated K263 on GAPDH. The results revealed that butyric acid treatment significantly increased K263 butyrylation while reducing lactylation at the same site. Correspondingly, the expression of calcification markers Runx2 and BMP2 was also significantly reduced (Figure 5M).

*In vivo*, oral administration of 600 mg/kg butyric acid in HF diet‐fed mice yielded similar results (Figure S11A–M). Notably, GAPDH K263 butyrylation inversely correlated with calcification severity, while lactylation at the same residue demonstrated a direct positive association with disease progression. (Figure S11N). Finally, hVICs isolated from eight healthy and eight calcified patients (Table S3) were examined. In‐cell western blot assays showed that GAPDH K263 butyrylation was significantly reduced, whereas the K263 lactylation was significantly increased in calcified valves. Both were significantly correlated with Runx2 and BMP2 expression (Figures 5N,O, S12).

Together, these results reveal a novel mechanism by which butyric acid inhibits aortic valve calcification by competitively promoting GAPDH K263 butyrylation, thereby blocking pro‐calcific GAPDH K263 lactylation.

## DISCUSSION

The gut microbiota, as a critical environmental factor, plays a pivotal role in the pathogenesis of various CVD. However, the specific mechanisms by which intestinal microbial dysbiosis influences the development and progression of CAVD remain poorly understood, and its clinical relevance has yet to be fully characterized. This study is the first to establish a close correlation between gut microbiota alterations and CAVD pathology. We identified the depletion of *F. prausnitzii* as a hallmark feature of CAVD progression and demonstrated that *F. prausnitzii*‐derived butyric acid is significantly reduced in the serum of CAVD patients, correlating strongly with CAVD risk. Furthermore, dietary supplementation with *F. prausnitzii* and butyric acid markedly attenuated valve calcification both in vitro and in vivo. Mechanistically, butyric acid exerts its anticalcific effects on hVICs by post‐translationally modulating the glycolytic pathway. Specifically, butyric acid occupies lysine residues via butyrylation, thereby blocking lactylation of the GAPDH protein, which ultimately mitigates CAVD progression.

In recent years, *F. prausnitzii*, which constitutes more than 5% of the human gut microbiota, has garnered substantial attention in preventing cardiovascular diseases [20]. *F. prausnitzii* has emerged as a universal, robust predictor of CVD and is strongly associated with various clinical characteristics [19]. Numerous studies have reported robust depletion of *F. prausnitzii* in the stool of patients with CVD, which is associated with markers of disease severity [21]. Notably, there was no difference in the abundance of *F. prausnitzii* between patients in disease remission and healthy controls. Consistent with these studies, we identified pronounced *F. prausnitzii* differences in the faeces of mice with difficult valve calcification compared with those of easily calcified mice. Dietary *F. prausnitzi*i supplementation significantly mitigated valve calcification‐related symptoms in ApoE^−/−^ mice. Furthermore, its supernatant can retard the osteogenic differentiation of hVICs. Numerous studies have demonstrated that the gut microbiota plays a crucial role in host health through the production of metabolites [22, 23]. Conversely, an imbalance or dysbiosis of the gut microbiota and its derived metabolites has been linked to various human diseases. Our study demonstrated that the altered serum metabolites were significantly correlated with both the abundance of *F. prausnitzii* and CAVD severity. These findings prompted us to hypothesize that microbial metabolites facilitate intricate host‐microbial interactions in CAVD.

Within the context of significant crosstalk between gut microorganisms and the cometabolism of compounds by the host and microbiota, a metabolite‐centric study design offers a functional perspective on host‐microbiota interactions. In the present study, we identified butyric acid as the metabolite most related to *F. prausnitzii*. Consistent with prior reports, butyric acid can be derived from *F. prausnitzii* [24]. The abundance of *F. prausnitzii* in faeces from mice prone to calcification is significantly reduced, accompanied by a striking decrease in the faecal and serum levels of butyric acid. This study further confirmed the critical role of *F. prausnitzii* in butyric acid production and demonstrated that reduced butyric acid levels advanced CAVD progression in ApoE^−/−^ mice. Moreover, our study in humans revealed that the serum butyric acid concentration was decreased in CAVD patients and closely associated with the clinical indices of aortic calcification, suggesting its promising prospects in the diagnosis of CAVD. In addition, the functional effects of butyric acid on CAVD remission were validated, and supplementation with butyric acid alleviated CAVD progression in ApoE^−/−^ mice. This is the first report to demonstrate that *F. prausnitzii* and *F. prausnitzii*‐derived metabolites exert potential protective effects against CAVD, which suggests that probiotic therapeutics may be amenable to clinical use for the treatment of CAVD in the future.

The reported effects of *F. prausnitzii*‐derived butyric acid on disease remission are diverse [25, 26, 27]. Despite numerous high‐quality associative studies examining the functional role of butyric acid in the regulation of cardiovascular disease, it remains unclear whether butyric acid influences the osteogenic differentiation of hVICs. Isotope labeling experiments and ^13^C flux analysis indicated that translocation of labeled butyric acid in the intestine into the bloodstream can shuttled to various organs and continue to reside there. The discovery of labeled butyric acid in cardiac tissue led us to propose that butyric acid might directly regulate the function and phenotype of hVICs to alleviate CAVD development. Consistent with this speculation, butyric acid strikingly dampened the osteogenic differentiation and inflammatory phenotype of hVICs. Although the receptors/targets for butyric acid have been increasingly decoded, how butyric acid‐initiated signaling pathways work holistically to fulfill the anti‐calcification effect remains enigmatic. To further explore the molecular mechanism by which butyric acid inhibits valve calcification, we conducted transcriptomic and metabolomic analyses of hVICs after butyric acid treatment. Multiomics analysis revealed that the anticalcification effect of butyric acid is linked to its systemic regulatory effect on glycolytic metabolism. Furthermore, seahorse assays verified that the ECAR and glycolytic ATP production rates significantly decreased after treatment with butyric acid. Overall, the findings of our study identified a novel regulatory role of butyric acid in valve calcification disease.

The link between glycolytic metabolism and CAVD has been recognized previously [28]. Our previous research revealed that uncontrolled glycolysis or abnormally activated glycolysis is easily observed in the metabolism of interstitial cells in CAVD [29]. Lactate, a byproduct of glycolysis metabolism, has been demonstrated to significantly influence tumor progression, metastasis, and immune evasion through diverse mechanisms [30, 31, 32]. However, there is currently no clear direct evidence for the correlation between the lactate level and CAVD risk. Lactate was extremely increased in calcified patients compared with healthy controls (unpublished data), indicating that lactate might be a good candidate biomarker to predict CAVD. Recent studies have revealed a novel function for lactate as a substrate for a newly discovered posttranslational modification, Kla, which regulates different pathophysiological states [33]. Our previous studies confirmed that Kla levels in the aortic valves of patients with CAVD are increased and that targeting Kla can prevent CAVD progression [28]. Recent studies have revealed that butyrate not only functions as a histone deacetylase (HDAC) inhibitor but also serves as a unique histone acylation marker, directly rewriting the “open code” of chromatin to regulate gene expression [34, 35]. Considering butyrate's role in epigenetic modulation and the interplay between metabolism and epigenetic modifications [36], we hypothesize whether butyric acid regulates glycolysis through modulating lactylation. Our results showed that butyric acid dramatically inhibited not only lactate production but also Kla levels. Interestingly, butyric acid specifically suppresses Kla at the lysine 263 site of GAPDH, concurrently increasing butyrylation (Kbu) at the same GAPDH K263 site. Mechanistically, the competitive binding of Kbu and Kla at the GAPDH K263 site reduces GAPDH lactylation levels, thereby inhibiting abnormal glycolysis driven by lactylated GAPDH. These findings offer novel insights into the molecular mechanisms underlying the pathological cascade in CAVD.

This study has several limitations. First, clinical samples should be collected for further validation due to the differences in gut microbiota composition and metabolic pathways between mice and humans. Second, the study focused on serum butyric acid concentrations in healthy individuals and CAVD patients; however, future investigations should include measurements in populations with bicuspid aortic valves (BAV) to evaluate potential correlations between BAV pathophysiology and gut microbial metabolite dynamics. Third, the study did not explore the broader influence of butyric acid on alternative signaling pathway or protein modification. Future studies should investigate its multifaceted regulatory mechanisms to fully elucidate its therapeutic potential in CAVD pathology. Furthermore, the rigorous fluorescent labeling or isotopic tracer studies should be conducted to validate that microorganisms are ingested orally and colonized in the intestine, rather than through skin contact or inhalation. Additionally, this study has demonstrated the potential therapeutic effects of *F. prausnitzii* and its metabolite butyrate in CAVD; however, their clinical application still faces critical challenges. Future research needs to evaluate the metabolic stability of orally administered *F. prausnitzii* or butyrate, and develop acid‐resistant microcapsules or sustained‐release formulations to ensure their localized action in the colon. Moreover, the optimal dosage and safety profile must be determined through dose‐escalation trials and long‐term toxicity studies to avoid potential adverse effects.

## CONCLUSION

In conclusion, we identified *F. prausnitzii*, a key gut microbiota, as being negatively associated with CAVD risk. *F. prausnitzii* slows the osteogenic differentiation of hVICs and decelerates the progression of CAVD through the previously undescribed metabolite butyric acid/epigenetic reshaping/glycolysis pathway. Furthermore, the present study demonstrated that *F. prausnitzii* and butyric acid are promising candidates for CAVD prevention and treatment.

## METHODS

### Human studies and ethics

Fifty blood samples from patients with CAVD (*n* = 25) and non‐CAVD (*n* = 25) patients were obtained from Union Hospital, Tongji Medical College, Huazhong University of Science and Technology. Human hVICs were acquired from noncalcified aortic valve specimens. The investigation conforms to the principles outlined in the Declaration of Helsinki and was approved by the Tongji Medical College Institutional Review Board, Huazhong University of Science and Technology (Approval No. [2024](0810)). Patient consent was obtained before the experiments.

### High‐fat diet‐induced heart valve calcification in ApoE^−/−^ mice

All the animal experiments were performed according to the instructions of the Ethics Committee of Hubei University of Chinese Medicine (Approval No. HUCMS 202304057). All procedures conform to the guidelines of the NIH Guide for the Care and Use of Laboratory Animals. SPF ApoE^−/−^ mice (3 weeks old) were purchased from the Experimental Animal Center of Huazhong University of Science and Technology (Wuhan, China), laboratory animal production license number: SCXK(e)2021‐009. The ApoE^−/−^ mice were fed a HF diet (42% fat, 0.25% cholesterol) for 26 weeks to induce aortic valve calcification. Single‐cage feed was used for each mouse, and the cages were refreshed every 2 weeks. After 26 weeks, the blood flow velocity and transvalvular pressure difference at the heart valves of all ApoE^−/−^ mice were detected via ultrasound examination, after which the heart valves were harvested for histological evaluation. Mouse anesthesia was induced with 3% isoflurane in an induction chamber. Maintenance anesthesia consisted of 1.5%–2% isoflurane delivered via a mask. The animals were euthanized via CO_2_ inhalation for sacrifice. All valves were subsequently cut to a thickness of 5 μm. Finally, Von Kossa and Masson staining were applied to evaluate the level of cardiac valve calcification according to the manufacturer's instructions. The oral butyric acid group was fed from week 14 to week 26 at a dose of 600 mg/kg.

### Dirty cage experiments and FMT

Seventy‐two ApoE^−/−^ mice were randomly selected from a large number of mice, divided into three groups (A, B, C) of 24 mice each (A1–A24, B1–B24, C1–C24), and subjected to a high‐fat diet in a single cage. For the dirty cage experiment, the mice in the high‐fat diet group (Group A) were moved to new cages every 2 weeks, and then, the mice in the dirty cage experimental group (Group B) were placed and reared in the dirty cages of the Group A mice. This process was repeated every 2 weeks, and shuffling ensured that the numbers matched. For the FMT experiment, faeces were collected from the mice in Group A every 2 days, and the faecal microbiota was transplanted into Group C, again ensuring that the numbers matched. The faecal transplantation experiment was performed as follows:

Before the experiment, donor mice (Group A) were co‐housed with recipient mice (Group C) for 1 week to standardize the initial microbiota through shared environmental exposure. Subsequently, Group C underwent a 10‐day pretreatment with a broad‐spectrum antibiotic regimen in drinking water (1.0 g/L ampicillin, 0.5 g/L vancomycin, 1.0 g/L neomycin, and 0.5 g/L metronidazole) to eliminate the original microbiota. During the transplantation period, donor faecal pathogens (*Clostridium difficile* and pathogenic *Escherichia coli*) were regularly monitored to ensure that effects were attributable to the transfer of live bacteria. Fresh faeces from Group A were collected under strict anaerobic conditions (85% N_2_, 10% H_2_, 5% CO_2_) to maintain microbial viability. The suspension was prepared at a standardized concentration, where 100 mg of faeces was accurately weighed and homogenized with pre‐reduced PBS (containing 0.05% l‐cysteine) to achieve a concentration of 100 mg/mL. The mixture was then centrifuged (500 *g* for 5 min) to remove larger particulate matter, and the supernatant was filtered through a 70 μm cell strainer and aliquoted into anaerobic tubes for storage (≥1 × 10⁸ CFU/mL). Group C received 200 μL of the bacterial suspension via oral gavage every 48 h for a total duration of 21 days. Moreover, 24 ApoE^−/−^ mice were fed a high‐fat diet, and the cages were changed every 2 days to ensure a clean rearing environment. After 26 weeks, the mice were randomly divided into two groups: Group B (Normal) and Group C Control (Ctrl). After 26 weeks, cardiac ultrasound experiments were performed, and the hearts of all the mice were harvested and histologically stained with Von Kossa and Masson stains.

### Group A mice were divided into easy and difficult calcification groups

PCA and OPLS‐DA were performed to classify the A‐groups into easy and difficult calcification groups by importing data based on Von Kossa staining (IOD), valve thickness, flow velocity (mm/s) and trans‐valve differential pressure (mmHg) of aortic valves in ApoE^−^^/^^−^ mice into https://new.metaboanalyst.ca/ModuleView.xhtml. After clustering, a confusion matrix and a fully connected neural network analysis were used to verify the above cluster. Briefly, load the data, separate the features and labels, and normalize the features to ensure that the features are on the same scale, which helps the training effect of the model. Secondly, divide the data set, divide the resulting data set into training set and validation set to ensure that the training and validation process of the model consists of good generalization ability. The ratio of training sets and validation sets is set to 80% and 20%. The proposed fully connected neural network model was constructed based on Pytorch (version 2.4.1). The network model contains two hidden layers, each with ReLU activation function, the size of the output layer is equal to the number of categories, and Mseloss is used for loss calculation. Training is performed by Adam optimizer with learning rate set to 0.001 and training number of generations set to 200. In each epoch, the training set is traversed to compute the loss and back propagation is performed to update the model parameters. After each epoch, the model performance is evaluated by calculating the accuracy of the validation set.

### Isolation and culture of human VICs

The hVICs were harvested from noncalcified aortic valve specimens according to our previous study [37]. After being washed three times in sterile PBS, the aortic valves were cut into small pieces and digested in 1 mg/mL type I collagenase for 8 h at 37°C. All the suspensions were subsequently collected, and undigested tissue was removed via a 70 µm nylon cell filter. The isolated cells were cultured in high‐glucose Dulbecco's modified Eagle's medium (DMEM) supplemented with 10% fetal bovine serum (FBS) and 1% penicillin‐streptomycin at 37°C in an atmosphere with 5% CO_2_. Cells from the third passage were utilized for subsequent experiments.

### 16S rRNA sequencing

Faeces from difficult calcification and easy calcification mice were collected and sent to Shanghai OE Biotech Co., Ltd for 16S rRNA gene sequencing. Total genomic DNA was extracted using MagPure Soil DNA LQ Kit (Magan) following the manufacturer's instructions. Sequencing was performed on an Illumina NovaSeq. 6000 with 250 bp paired‐end reads. The library sequencing and data processing were conducted by OE Biotech Co., Ltd. Raw sequencing data were in FASTQ format. Paired‐end reads were then preprocessed using Cutadapt software (Version 5.0) to detect and cut off the adapter. After trimming, paired‐end reads were filtering low‐quality sequences, denoised, merged and detected and cut off the chimera reads using DADA2 [38] with the default parameters of QIIME2 [39] (https://qiime2.org/). At last, the software outputs the representative reads and the ASV abundance table. The representative read of each ASV was selected using QIIME2 package. All representative reads were annotated and blasted against Silva database (Version 138) using q2‐feature‐classifier with the default parameters. QIIME2 software was used for alpha and beta diversity analysis. The microbial diversity in samples was estimated using the alpha diversity, which includes Chao1 index and Shannon index. The unweighted Unifrac distance matrix performed by R package (Version 3.5.1) was used for unweighted Unifrac Principal coordinates analysis (PCoA) to estimate the beta diversity. Then the R package (Version 3.5.1) was used to analyze the significant differences between different groups using *t*‐test statistical test. The linear discriminant analysis effect size (LEfSe) method was used to compare the taxonomy abundance spectrum. The LEfSe method was used to compare the taxonomy abundance spectrum. Top 10 species were selected based on relative abundance. The species could be detected in each sample were adopted for further analyses.

### Nontargeted metabolomics analysis

The metabolomic data analysis was performed by Shanghai Luming Biological Technology Co., Ltd. An ACQUITY UPLC I‐Class plus (Waters Corporation, Milford, USA) fitted with Q‐Exactive mass spectrometer equipped with heated electrospray ionization (ESI) source (Thermo Fisher Scientific) was used to analyze the metabolic profiling in both ESI positive and ESI negative ion modes. An ACQUITY UPLC HSS T3 column (1.8 μm, 2.1 × 100 mm) were employed in both positive and negative modes. The mass range was from m/z 100 to 1000. The resolution was set at 70,000 for the full MS scans and 17500 for HCD MS/MS scans. The Collision energy was set at 10, 20 and 40 eV. The mass spectrometer operated as follows: spray voltage, 3800 V (+) and 3200 V (−); sheath gas flow rate, 35 arbitrary units; auxiliary gas flow rate, eight arbitrary units; capillary temperature, 320°C; Aux gas heater temperature, 350°C; S‐lens RF level, 50. The LC‐MS data were processed by software Progenesis QI V2.3 (Nonlinear Dynamics) for baseline filtering, peak identification, integral, retention time correction, peak alignment, and normalization. A data matrix was combined from the positive and negative ion data, which was imported in https://new.metaboanalyst.ca/ModuleView.xhtml to carry out PCA and OPLS‐DA. PCA were utilized to observe the overall distribution among the samples and the stability of the whole analysis process. OPLS‐DA were utilized to distinguish the metabolites that differ between groups. Variable Importance of Projection (VIP) values obtained from the OPLS‐DA model were used to rank the overall contribution of each variable to group discrimination. A two‐tailed Student's *t* test was further used to verify whether the metabolites of difference between groups were significant. Differential metabolites were selected with VIP values greater than 1.0 and *p* values less than 0.05. In addition, after Venn of metabolites, and further final common fat acid (FA) metabolites were selected. The Pearson correlation analysis was performed based on the above final common FA metabolites and the selected species.

### *F. prausnitzii* transplantation and cell/tissue culture

*F. prausnitzii* was purchased from Bei Na Bio (BNCC), source number: ATCC 27768. Reinforced medium for clostridium (RCM) was used to cultivate *F. prausnitzii* in an anaerobic environment at 37°C. After harvesting, the bacterial concentration was adjusted to 2.5 × 10^9^ cfu/100 μL, and the prepared bacterial mixture was transferred to an anaerobic tube in a standby state for backup. ApoE^−/−^ mice were maintained under specific pathogen‐free conditions in individually ventilated cages (IVCs) and administered a broad‐spectrum antibiotic cocktail (containing 1.0 g/L ampicillin, 0.5 g/L vancomycin, 1.0 g/L neomycin, and 0.5 g/L metronidazole) via drinking water for 10 consecutive days to deplete Gram‐positive bacteria (targeted by vancomycin), Gram‐negative bacteria (targeted by ampicillin/neomycin), and anaerobic bacteria (targeted by metronidazole), thereby establishing a gut microbiota‐depleted model. The antibiotic solution was prepared fresh daily and administered in light‐protected bottles. These microbiota‐depleted mice were then randomly divided into two groups: Control (antibiotics + PBS) and *F. prausnitzii*‐treated (FPT). From day 11 onward, the Control and FPT groups received daily oral gavage of 100 μL sterile PBS or *F. prausnitzii* suspension (2.5 × 10⁹ cfu/100 μL), respectively, and were maintained on a high‐fat diet for 21 days. After completing the strain transplantation, the high‐fat diet was continued for up to 26 weeks, and during this period, three bacterial harvests were carried out to replenish the strains. Sera from mice after *F. prausnitzii* single‐bacteria transplantation were collected, and hVICs in the calcification‐induced state were treated with a final concentration of 10%. The medium supernatant of *F. prausnitzii* (FPM) with a bacterial concentration of 2.5 × 10^9^ cfu/100 μL was collected and subsequently subjected to centrifugation to remove the bacteria. The resulting sterile supernatant was then processed by microporous filter membrane filtration and added to the cell culture medium at various ratios to intervene in the osteogenic differentiation of hVICs induced by OM. The ex vivo cultured valve tissues were treated with a final concentration of 10% *F. prausnitzii* to detect the expression of BMP2 and Runx2.

### Gas chromatography‐mass spectrometry (GC‐MS) analysis of ^13^C‐labeled butyric acid tracers

After continuous oral administration of ^13^C‐labeled butyric acid at a dose of 600 mg/kg in a mouse model of aortic valve calcification, mouse faeces were collected, and the serum and corresponding tissue samples were stored at −80°C. Fifty milligrams of the corresponding tissue samples were weighed, 0.2 mL of 0.5% aqueous phosphoric acid solution was added, the samples were ground twice for 15 s each time in a 20 Hz ball mill, vortexed and shaken for 10 min to mix well. Then, 500 μL of tert‐butyl methyl ether was added, vortexed, mixed well, and sonicated in an ice bath for 5 min. The sample was then centrifuged at 12,000 rpm (Centrifuge 5418R, Eppendorf, Germany) for 10 min at 4°C, and the supernatant was extracted into 200 μL, filtered through a 0.22 μm microporous membrane, and then stored at −20°C for further online analyses. The following standards were prepared with tert‐butyl methyl ether as the solvent: n‐butyric acid (0.048 mg/mL), ^13^C‐labeled butyric acid (0.046 mg/mL), and a DB‐FFAP capillary column (Agilent Technologies, USA). Mass spectral data were obtained in full scan mode with mass‐charge ratios (m/z) in the range of 20–100. GC‐MS data were analyzed via Xcalibur software.

### Ex vivo osteogenic differentiation

The effects of butyric acid on the OM‐induced calcification of hVICs were detected through the alizarin red staining test. The hVICs were seeded on a 35 mm‐diameter dish at a density of 3 × 10^3^ cells/cm^2^ in DMEM supplemented with 10% FBS. After cell starvation in DMEM supplemented with 2% FBS, hVICs were further incubated with OM comprising 10 mM β‐glycerol phosphate disodium salt pentahydrate, 50 μg/mL vitamin C and 0.1 μM dexamethasone. Three groups were established in this study, including the control, OM, and BA groups. The OM was replenished every 2 days, and butyric acid was added to the OM in the BA group. Alizarin red staining was performed to detect calcium deposition after continuous culture for 23 days, and Runx2 and BMP2 expression were verified by in‐cell western blot analysis. Simultaneously, RNA‐seq and metabolomics analyses were performed to verify the changes in mRNA expression and cellular substance metabolites. Message RNA sequencing (mRNA‐seq) quantitation was used to analyze the changes in cellular mRNA profiles between treatments. Cells were harvested using Trizol reagent (Vazyme, Nanjing, China) to isolate total RNA. The RNA samples reached A class quality (RQN > 7.0; 28S/18S > 1.0) were sent to BGI Ltd. (Wuhan, China) for RNA sequencing. Non‐targeted metabolomics analysis was performed with GC‐MS platform. Briefly, cells were collected in methanol on ice and were repeatedly freeze‐thawed three times with liquid nitrogen. After centrifugation at 12,000 rpm for 10 min at 4°C, the supernatants were harvested. The harvested samples were then blown dry with nitrogen, then mixed with methoxypyridine solution (20 mg/mL) and of BSTFA (sigma). After incubation in a water bath at 80°C for 1 h, the supernatant was separated by centrifugation at 12,000 rpm for 10 min at 4°C. The prepared samples were analyzed by Trace 1300 GC‐MS system. Moreover, human aortic valve leaflets were harvested from patients undergoing heart transplantation. These samples were cut into 2 mm × 2 mm pieces and exposed to OM for 23 days before the calcific levels were verified by detecting calcium deposition and Runx2 and BMP2 expression.

### Oral administration of butyric acid to ApoE^−/−^ mice

Butyric acid was applied to high‐fat‐fed ApoE^−/−^ mice via oral administration, and its effects on heart valve calcification were detected in vivo. High‐fat feeding was performed as described above, while 600 mg/kg butyric acid was administered orally to the HF + BA group once each day. Finally, Von Kossa staining and ultrasound were used to evaluate the level of cardiac valve calcification after the consumption of a high‐fat diet for 26 weeks.

### In‐cell Western

In‐cell western blot analysis was performed according to the manufacturer's instructions (CellTag 700 Stain ICW KIT). Briefly, cells were seeded into black 96‐well WB plates under sterilized conditions, and when the coverage reached approximately 70%, 500 μM butyric acid was used to treat the cells for 48 h. After that, the conditioned medium was removed. Then, the cells were fixed with 4% PFA. After permeabilization with 0.1% Triton X‐100 PBS solution several times, LI‐COR blocking solution (100 μL) was added to each well and shaken at room temperature for 1 h. Additionally, the cells were incubated with primary antibodies overnight, and then secondary antibody dilutions were added and incubated at room temperature under dark light for 1 h. Finally, 0.1% Tween‐20 PBS solution was added to each well and washed five times, after which the liquid was aspirated and imaged on an Odyssey instrument system.

### Ultrasound detection

ApoE^−/−^ mice were fed a high‐fat diet (42% fat and 0.25% cholesterol) for 26 weeks to induce aortic valve calcification, with an oral butyric acid dose of 600 mg/kg from week 14 to week 26. Mouse aortic valve tissue samples were subsequently collected. Small animal ultrasound was used to evaluate peak pressure and flow velocity around the aortic valve.

### Ex vivo culture

Human aortic valve leaflets were cut into 2 mm × 2 mm pieces and treated with 500 μM butyric acid under OM induction conditions. After that, the ex vivo tissues were fixed with 4% PFA for 30 min and then embedded in paraffin to make 5‐μm‐thick sections. Immunofluorescence staining was used to detect the expression levels of related proteins in these samples. Then, calcification was evaluated via Von Kossa staining. Von Kossa intensity was measured via semiquantitative analysis via ImageJ software.

### Metabolic extracellular flux analysis

Real‐time changes in the ECAR of cells were determined via an XF‐96 Seahorse extracellular flux analyzer (Agilent Seahorse XF Technology). Briefly, hVICs were plated onto XF‐96 cell culture plates in the presence or absence of butyric acid under OM induction conditions. On the day of the assay, the cells were incubated in buffer solution for 2 h before the assay was performed in a non‐CO_2_ incubator at 37°C. The assay was initiated by sequential injections: first with glucose (10 mM), followed by oligomycin (Oligo), and finally 2‐deoxy‐D‐glucose (2‐DG). Glycolytic stress was assessed according to the manufacturer's protocol (Seahorse Bioscience).

### Cellular metabolomics analysis with butyric acid treatment

Cellular metabolomics analysis with butyric acid treatment was performed as non‐targeted metabolomics analysis based on GC‐MS platform. Briefly, cells were collected in methanol on ice and were repeatedly freeze‐thawed three times with liquid nitrogen. After centrifugation at 12,000 rpm for 10 min at 4°C, the supernatants were harvested. The harvested samples were then blown dry with nitrogen, then mixed with methoxypyridine solution (20 mg/mL) and of BSTFA (sigma). After incubation in a water bath at 80°C for 1 h, the supernatant was separated by centrifugation at 12,000 rpm for 10 min at 4°C. The prepared samples were analyzed by Trace 1300 GC‐MS system (Thermo).

### Immunoprecipitation

Immunoprecipitation (IP) was performed according to the manufacturer's instructions for the Immunoprecipitation Kit with Protein A + G Magnetic Beads (Beyotime). First, treated or untreated cells or valve tissue were lysed via IP lysis buffer. Next, the appropriate primary antibody or isotype control IgG was first incubated with Protein A+G magnetic beads to allow binding. The antibody‐bound beads were then added to the protein lysate at a ratio of 20 μL of bead suspension per 500 μL of sample and incubated overnight at 4°C on a rotary mixer. Finally, the protein sample were eluted with SDS‐PAGE buffer and analysed by Western blotting.

### Plasmid construction for site mutation

Primers for cloning the GAPDH open reading frame were synthesized by Wuhan Paivibio Biotechnology Co., Ltd. The forward primer (CMV‐F: 5′‐CGCAAATGGGGCGGGTAGGCGTG) and reverse primer (pCDH1‐R: 5′‐CCTTCTCTAGGCACCCGTTCAAT) were designed for insertion into the pCDH‐CMV‐MCS‐EF1α‐Puro vector. The kla at the sites 194/215/263 of GAPDH was mutated to arginine R (Arg) to mimic the delactylated state of this site. Site mutation plasmids were constructed and transfected into hVICs, and calcification‐related indicators were measured.

### Statistical analysis

The statistical analyses were conducted via SPSS 22.0 and GraphPad Prism 8.0 software. One‐way ANOVA was employed for multiple comparisons, whereas *t* tests were used for two‐group comparisons. Post hoc tests were only conducted when the F value reached a significance level of *p* < 0.05 and exhibited no significant inhomogeneity of variance. The analyzed data are presented as the means ± standard deviations (SDs), with a *p*‐value of less than 0.05 indicating a statistically significant difference.

## AUTHOR CONTRIBUTIONS

**Chunli Wang**: Conceptualization; supervision; investigation; project administration; writing—original draft; funding acquisition. **Zongtao Liu**: Formal analysis; data curation. **Tingwen Zhou**: Resources; software. **Jiaqin Wu**: Validation; formal analysis. **Fan Feng**: Formal analysis; methodology. **Shunshun Wang**: Software; resources. **Qingjia Chi**: Methodology; formal analysis. **Yongqiang Sha**: Visualization. **Shuai Zha**: Visualization. **Songren Shu**: Formal analysis. **Linghang Qu**: Validation. **Qianqian Du**: Data curation; methodology. **Huiming Yu**: Data curation; visualization. **Li Yang**: Resources. **Anna Malashicheva**: Supervision. **Nianguo Dong**: Project administration. **Fei Xie**: Writing—review and editing; methodology; software; data curation. **Guixue Wang**: Funding acquisition; project administration; supervision; visualization. **Kang Xu**: Conceptualization; project administration; funding acquisition; writing—review and editing; resources.

## CONFLICT OF INTEREST STATEMENT

The authors declare no conflicts of interest.

## ETHICS STATEMENT

The investigation conforms to the principles outlined in the Declaration of Helsinki and was approved by the Tongji Medical College Institutional Review Board, Huazhong University of Science and Technology (Approval No. [2024](0810)). All the animal experiments were performed according to the instructions of the Ethics Committee of Hubei University of Chinese Medicine (Approval No. HUCMS 202304057).

## Supporting information


**Figure S1.** Clustering analyses to group difficult calcification and easy calcification mice, related to Figure 1.
**Figure S2.** Sequencing of gut microbes in difficult calcification versus easy calcification mice, related to Figure 2.
**Figure S3.** *F. prausnitzii* regulates calcification in hVICs, related to Figure 2.
**Figure S4.** Metabolomic analysis of faeces and serum from ApoE^−/−^ mice with easy calcification and difficult calcification, related to Figure 3.
**Figure S5.** Butyric acid was significantly associated with the abundance of *F. prausnitzii*, related to Figure 3.
**Figure S6.** Butyric acid levels in the liver, spleen, lung and kidney of ApoE^−/−^ mice in different treatment groups were detected via GC‐MS, related to Figure 3.
**Figure S7.** The changes in butyric acid levels in the cardiac tissues of ApoE^−/−^ mice at different time intervals after the oral administration of ^13^C‐labelled butyric acid were analysed via GC‐MS, related to Figure 3.
**Figure S8.** Effects of butyric acid on cell viability and the mRNA expression levels of *Runx2* and *BMP2*, related to Figure 4.
**Figure S9.** Gene expression profiles of hVICs in the control, OM and BA groups, related to Figure 4.
**Figure S10.** Effect of butyric acid treatment on the level of lactylation of glycolytic enzymes in OM‐induced hVICs, related to Figure 5.
**Figure S11.** Butyric acid feeding attenuated valve calcification in high‐fat diet‐fed mice, related to Figure 5.
**Figure S12.** The hVICs were extracted from the valves of 8 calcified patients and 8 healthy patients for in‐cell WB detection of the calcification markers Runx2/BMP2 and GAPDH K263 butyrylation/lactylation, related to Figure 5.

**Table S1.** Human samples information related to Figure 3.
**Table S2.** Comparison of the lactylation profiles of normal hVICs and OM‐induced hVICs revealed that lactylation of glycolytic enzymes is involved in the process of valve calcification, related to Figure 5.
**Table S3.** Human samples information related to Figure 5.

## Data Availability

The data that support the findings of this study are available from the corresponding author upon reasonable request. The 16S rRNA data (PRJNA1166408) linking to https://www.ncbi.nlm.nih.gov/bioproject/PRJNA1166408 and RNA‐sequencing data (PRJNA1173845) linking to https://www.ncbi.nlm.nih.gov/bioproject/PRJNA1173845 can be accessed in the Sequence Read Archive (SRA) database of NCBI. The data and scripts used are saved in GitHub https://github.com/Feng-19942025/iMeta-2025-061.git. Supplementary materials (figures, tables, graphical abstract, slides, videos, Chinese translated version and update materials) may be found in the online DOI or iMeta Science http://www.imeta.science/.
