# Supplementary material for: Gut microbiota‐derived butyric acid regulates calcific aortic valve disease pathogenesis by modulating GAPDH lactylation and butyrylation
Source: Imeta. 2025 May 19;4(4):e70048. doi: 10.1002/imt2.70048 (PMC12371252; doi:10.1002/imt2.70048)
Supplement: Supplementary file 1 — Figure S1. Clustering analyses to group difficult calcification and easy calcification mice, related to Figure 1. Figure S2. Sequencing of gut microbes in difficult calcification versus easy calcification mice, related to Figure 2. Figure S3. F. prausnitzii regulates calcification in hVICs, related to Figure 2. Figure S4. Metabolomic analysis of faeces and serum from ApoE−/− mice with easy calcification and difficult calcification, related to Figure 3. Figure S5. Butyric acid was significantly associated with the abundance of F. prausnitzii, related to Figure 3. Figure S6. Butyric acid levels in the liver, spleen, lung and kidney of ApoE−/− mice in different treatment groups were detected via GC‐MS, related to Figure 3. Figure S7. The changes in butyric acid levels in the cardiac tissues of ApoE−/− mice at different time intervals after the oral administration of 13C‐labelled butyric acid were analysed via GC‐MS, related to Figure 3. Figure S8. Effects of butyric acid on cell viability and the mRNA expression levels of Runx2 and BMP2, related to Figure 4. Figure S9. Gene expression profiles of hVICs in the control, OM and BA groups, related to Figure 4. Figure S10. Effect of butyric acid treatment on the level of lactylation of glycolytic enzymes in OM‐induced hVICs, related to Figure 5. Figure S11. Butyric acid feeding attenuated valve calcification in high‐fat diet‐fed mice, related to Figure 5. Figure S12. The hVICs were extracted from the valves of 8 calcified patients and 8 healthy patients for in‐cell WB detection of the calcification markers Runx2/BMP2 and GAPDH K263 butyrylation/lactylation, related to Figure 5. [file IMT2-4-e70048-s001.docx]

**Supporting information to**

# Gut microbially produced butyric acid impacts calcific aortic valve disease pathogenesis by mediating competitive modification of lactylation and butyrylation

**Running title: Gut microbiota regulates calcific aortic valve disease**

Chunli Wang ^1,2#^*, Zongtao Liu ^3#^, Tingwen Zhou ^3#^, Jiaqin Wu ^1,2#^, Fan Feng ^1,4#^, Shunshun Wang ^4^, Qingjia Chi ^5^, Yongqiang Sha ^6^, Shuai Zha ^1,2^, Songren Shu ^7^, Linghang Qu ^1,4^, Qianqian Du ^4^, Huiming Yu ^4^, Li Yang ^8^, Anna Malashicheva ^9^, Nianguo Dong ^3^*, Fei Xie ^10^*, Guixue Wang ^8,11^*, Kang Xu ^1,4^*

1 Hubei Shizhen Laboratory, Wuhan 430065, China.

2 School of Laboratory Medicine, Hubei University of Chinese Medicine, Wuhan 430065, China.

3 Department of Cardiovascular Surgery, Union Hospital, Tongji Medical College, Huazhong University of Science and Technology, Wuhan 430022, China.

4 School of Pharmacy, Hubei University of Chinese Medicine, Wuhan 430065, China.

5 College of Electrical Engineering and Automation, Anhui University, Hefei 230601‌‌, China.

6 Center for Precision Medicine, School of Medicine and School of Biomedical Sciences, Huaqiao University, Xiamen 361021, China.

7 Fuwai Hospital, National Center for Cardiovascular Diseases, Chinese Academy of Medical Sciences and Peking Union Medical College, Beijing 100037‌‌, China.

8 Ministry of Education Key Laboratory for Biorheological Science and Technology, National Local Joint Engineering Lab for Vascular Implants, College of Bioengineering, Chongqing University, Chongqing 400044, China.

9 Institute of Cytology, Russian Academy of Science, Petersburg 194064, Russia.

10 Department of Cardiovascular Surgery, The First Affiliated Hospital of Zhengzhou University, Zhengzhou 450052, China.

11 JinFeng Laboratory, Chongqing 401329, China.

^#^These authors contributed equally: Chunli Wang, Zongtao Liu, Tingwen Zhou, Jiaqin Wu, Fan Feng

*Correspondence: lilywang@cqu.edu.cn (Chunli Wang), dongnianguo@hotmail.com (Nianguo Dong), xiefei0103@163.com (Fei Xie), wanggx@cqu.edu.cn (Guixue Wang), kangxu05@hbucm.edu.cn (Kang Xu)


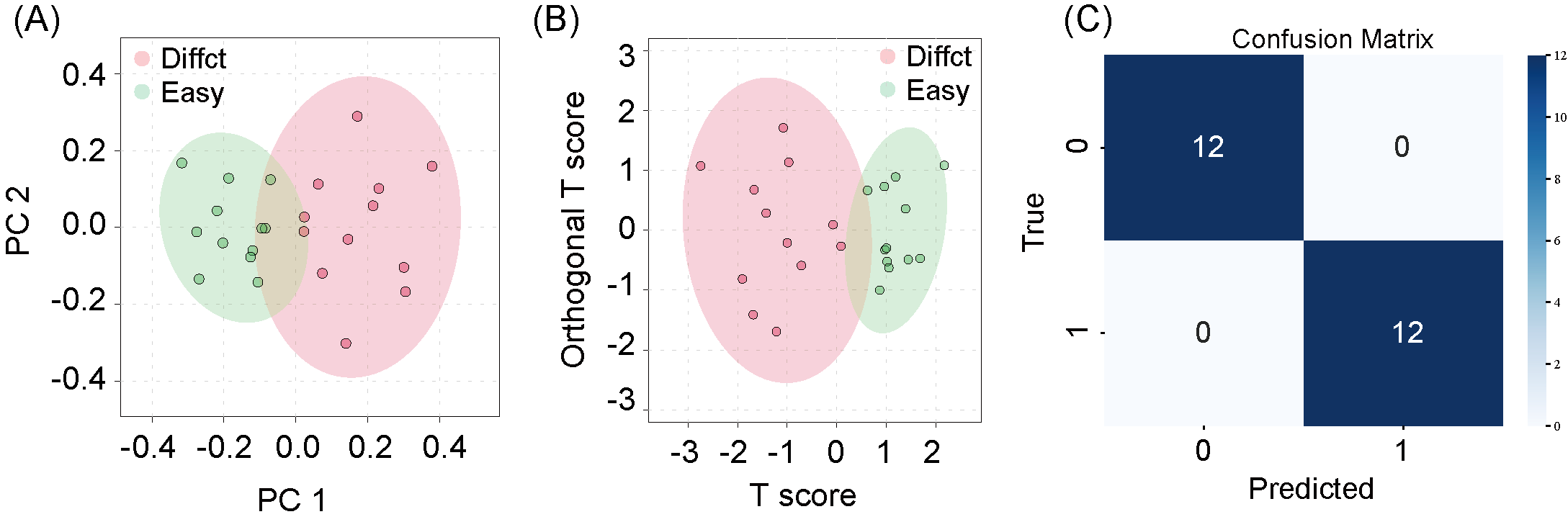


**Figure S1 Clustering analyses to group difficult calcification and easy calcification mice on the basis of Von Kossa staining (IOD) of mouse aortic valves, valve thickness, flow velocity (mm/s) and transvalvular pressure difference (mmHg).** (A) PCA cluster analysis. (B) OPLS-DA cluster analysis. (C) Neural network analysis.


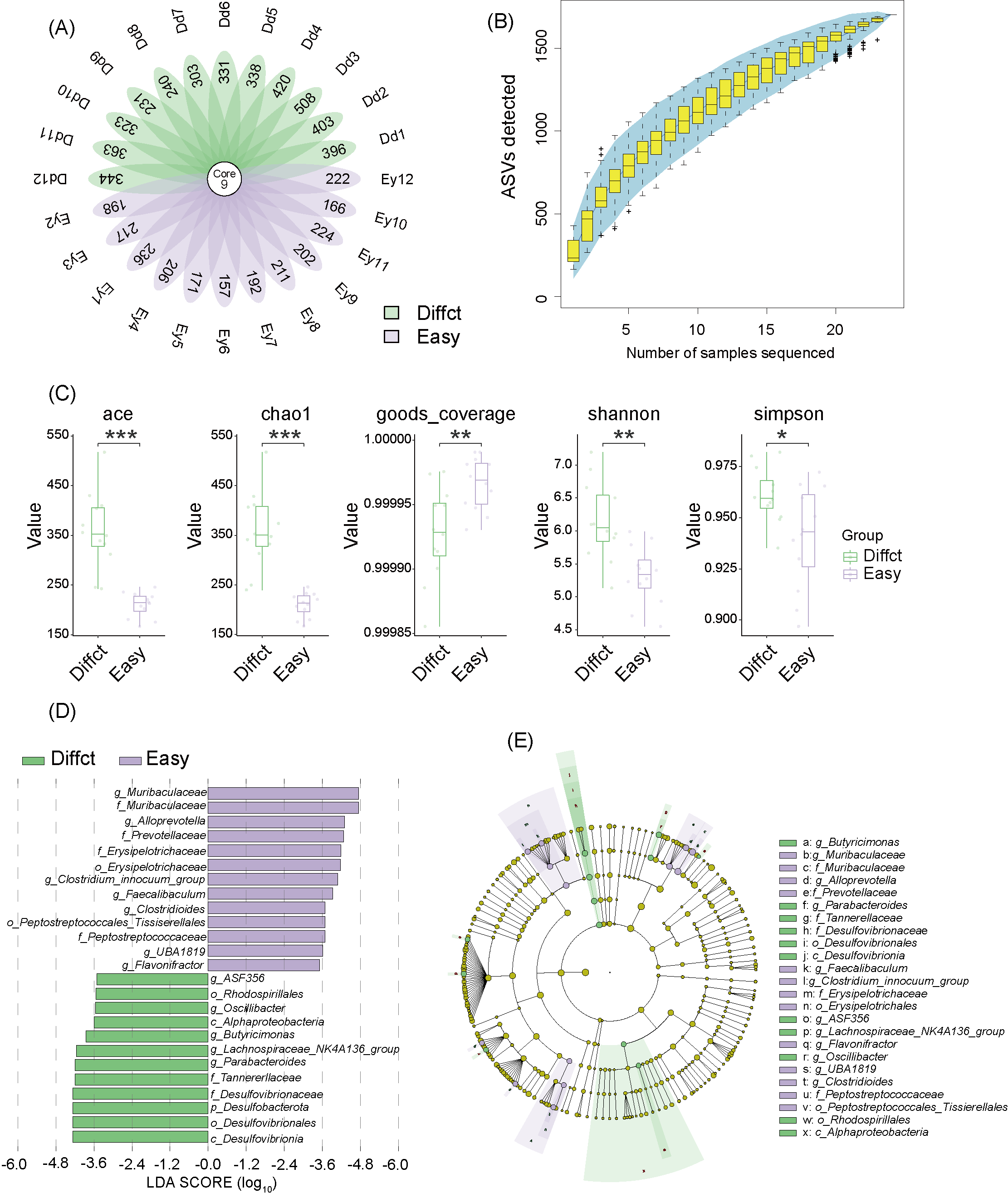


**Figure S2 Sequencing of gut microbes in difficult calcification (Diffct) versus easy calcification (Easy) mice.** (A) The numbers in the cores in the petal plots represent the number of ASVs common to all the samples (core ASVs), and the numbers in the petals represent the number of total ASVs minus the number of shared ASVs in each sample. (B) Specaccum species cumulative curves. (C) Sample alpha diversity analysis. (D) Differential species LDA score plot. (E) Example plot of annotated branches of differential species.


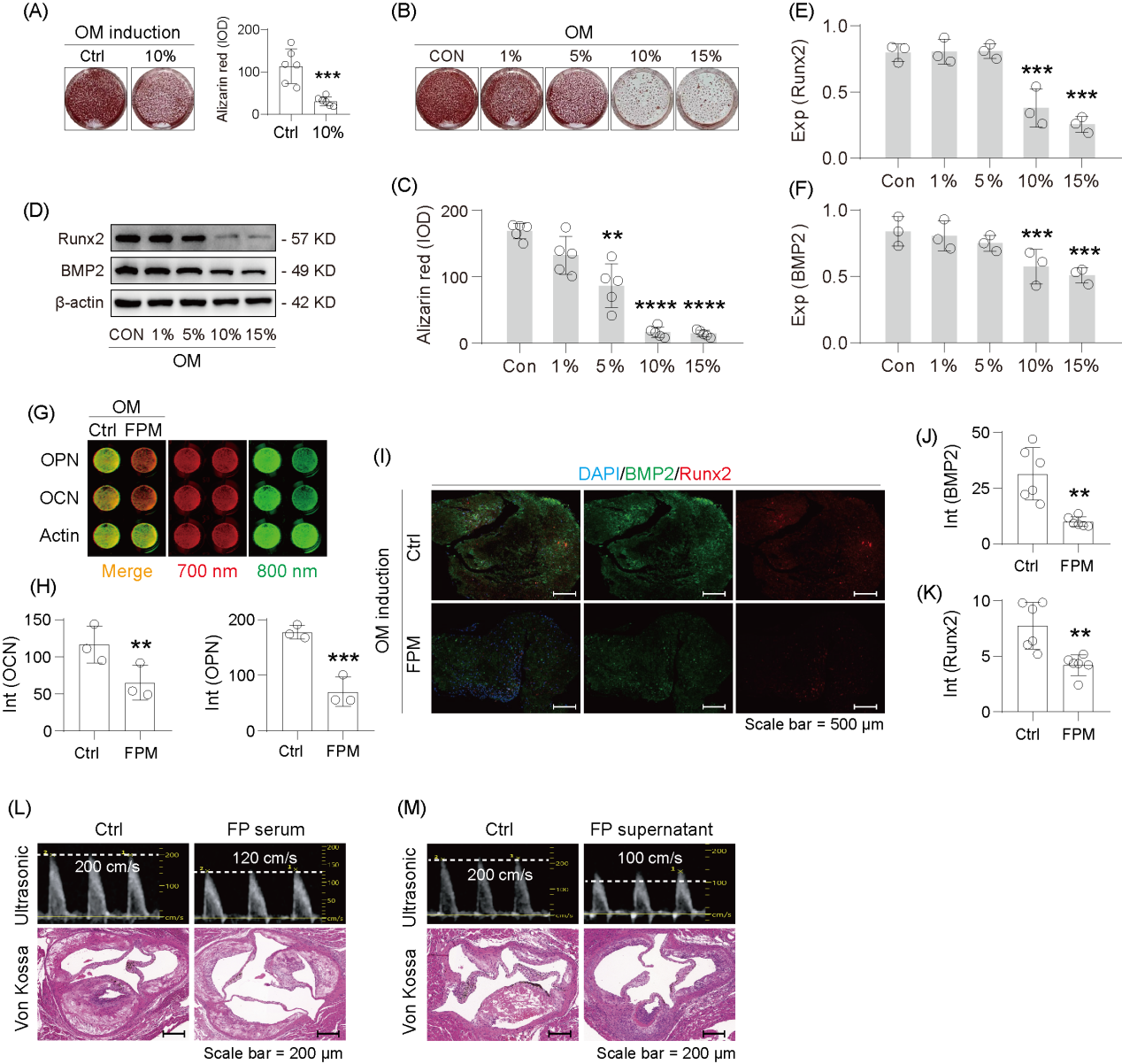


**Figure S3 *F. prausnitzii* regulates calcification in hVICs.** (A) Mouse serum was taken from *F. prausnitzii* after single bacterial transplantation, and hVICs in the calcification-induced state were treated with 10% final concentration. (B-H) *F. prausnitzii* supernatant medium (FPM) of *F. prausnitzii* was taken, and hVICs in the calcification-induced state were treated with 1−15% final concentration, which were screened to determine the optimal bacterial fluid concentration of 10% by alizarin red staining (B and C), Runx2 and BMP2 protein expression levels (D-F), OPN and OCN expression levels (G and H), and the optimal bacteriophage concentration of 10% was determined by screening. (I-K) *Ex vivo* cultured valve tissues were treated with 10% final concentration of *F. prausnitzii* solution to detect the expression of BMP2 and Runx2; scale bar: 500 μm. (L and M) The histological evaluation of ApoE^-/-^ mice with the treatment of the serum from the mice transplanted with *F. prausnitzii* (L), and from the supernatant from the culturing of *F. prausnitzii* (M). **p* < 0.05, ***p* < 0.01, ****p* < 0.001 indicate significant differences compared with the control group.


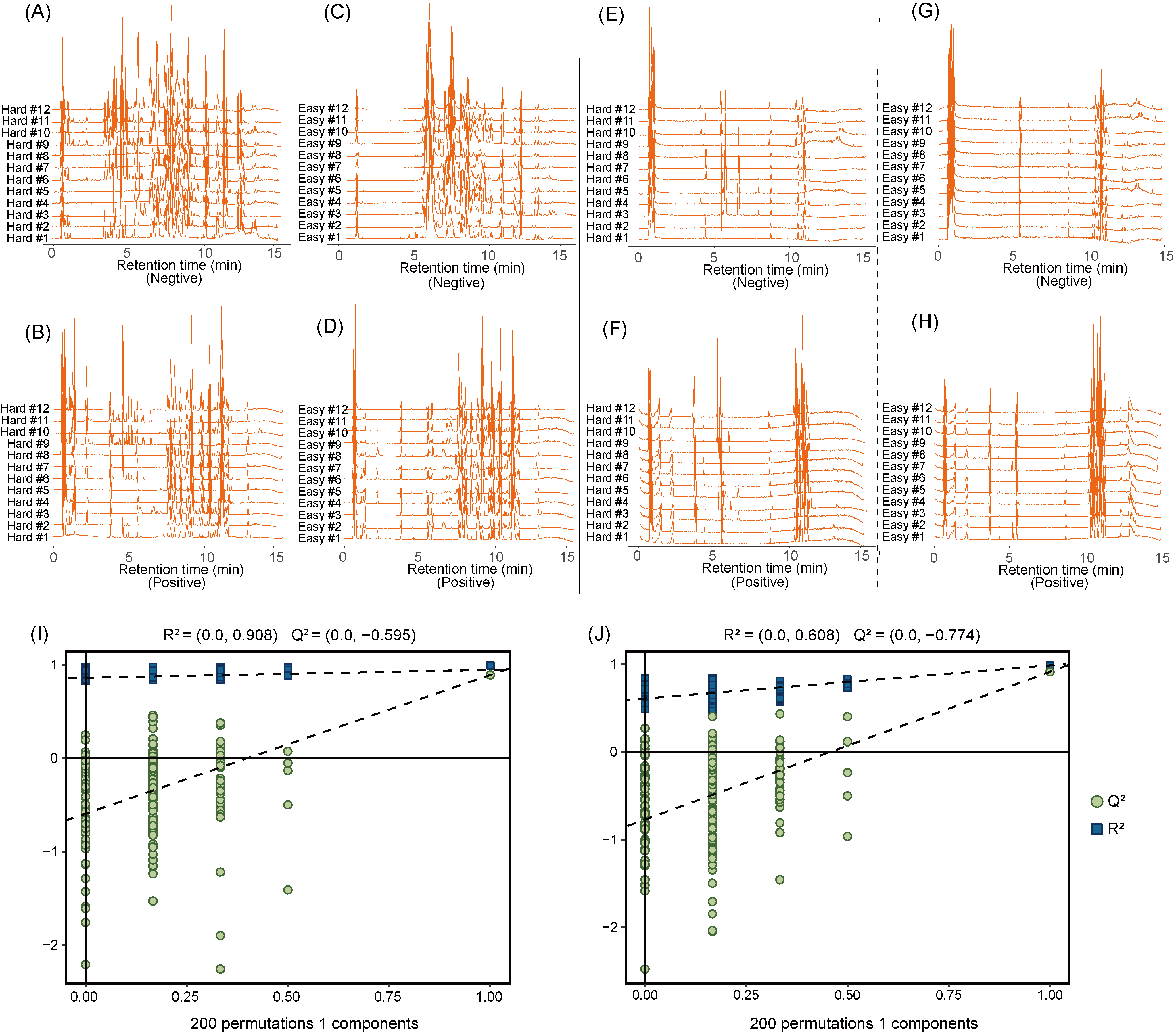


**Figure S4 Metabolomic analysis of faeces and serum from ApoE^-/-^ mice with easy calcification and difficult calcification.** (A and B) Base peak chromatograms of metabolites susceptible to calcification and refractory to faeces from difficult-calcification ApoE^-/-^ mice in positive- and negative-ion models. (C and D) Base peak chromatograms of metabolites susceptible to calcification and refractory to faeces from easy-calcification ApoE^-/-^ mice in positive- and negative-ion models. (E and F) Base peak chromatograms of metabolites susceptible to calcification and refractory to serum from difficult-calcification ApoE^-/-^ mice in positive- and negative-ion models. (G and H) Base peak chromatograms of metabolites susceptible to calcification and refractory to serum from easy-calcification ApoE^-/-^ mice in positive- and negative-ion models. (I and J) Model validation results in SIMCA for susceptible mouse faeces and serum, which showed R² = (0.0, 0.908) and Q² = (0.0, -0.595) and R² = (0.0, 0.608 and Q² = (0.0, -0.774).

**
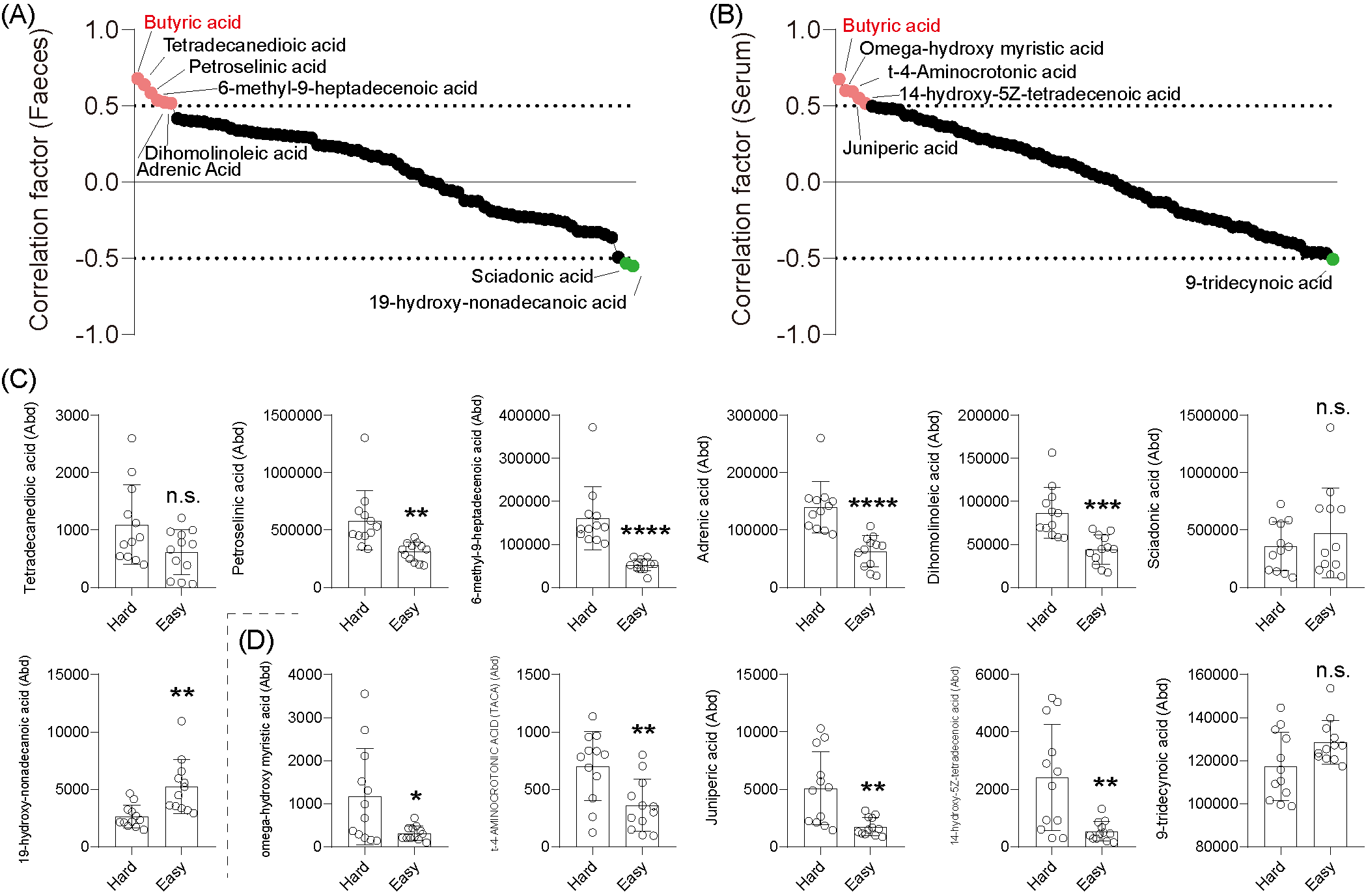
**

**Figure S5 Butyric acid was significantly associated with the abundance of *F. prausnitzii*.** (A and B) Correlation analysis of faecal and serum co-differentiated metabolites with *F. prausnitzii* abundance in easy-calcification and difficult-calcification ApoE^-/-^ mice. (C) Statistical analysis of the peak areas of major differentially abundant metabolites in the faeces of easy-calcification and difficult-calcification ApoE^-/-^ mice. (D) Statistical analysis of the peak areas of major differentially abundant metabolites in the sera of easy-calcification and difficult-calcification ApoE^-/-^ mice. **p* < 0.05, ***p* < 0.01, ****p* < 0.001 indicate significant differences compared with the control group.


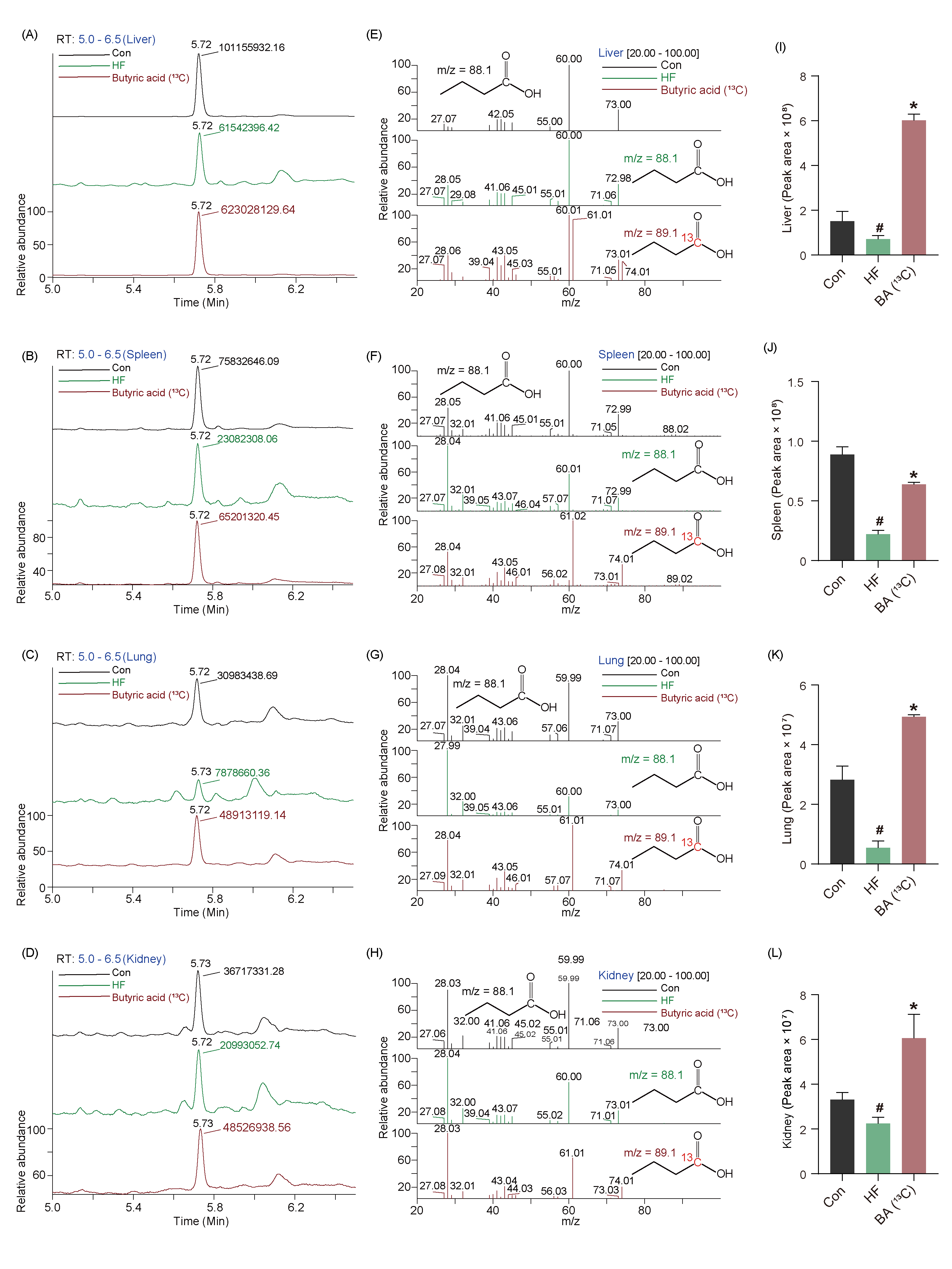


**Figure S6 Butyric acid levels in the liver, spleen, lung and kidney of ApoE^-/-^ mice in different treatment groups were detected via GC-MS.** (A-D) TIC flow diagram of butyric acid in the liver, spleen, lung and kidney of ApoE^-/-^ mice in different treatment groups. (E-H) MS analysis of full MS mode of butyric acid in the liver, spleen, lung and kidney of ApoE^-/-^ mice in different treatment groups. (I-L) Analysis of the butyric acid peak area in the liver, spleen, lung and kidney of ApoE^-/-^ mice. **p <* 0.05 and ^#^*p* indicate a significant difference.

**
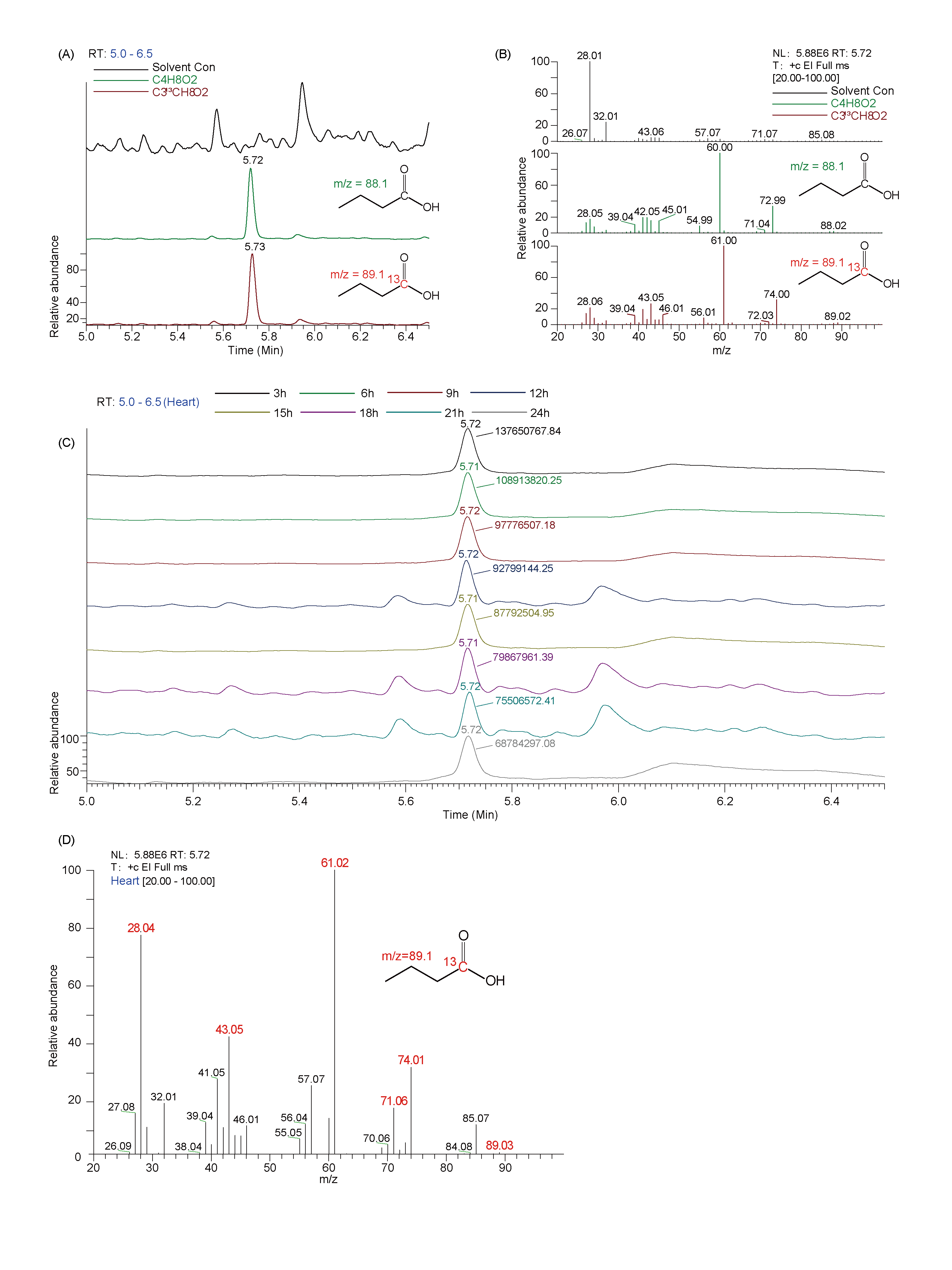
Figure S7 The changes in butyric acid levels in the cardiac tissues of ApoE^-/-^ mice at different time intervals after the oral administration of ^13^C-labelled butyric acid were analysed via GC-MS.** (A and B) Ion flow diagram and MS analysis of the full MS mode of different butyric acid standards. Solvent Con: solvent standard; C_4_H_8_O_2_: butyric acid standard; C_3_^13^CH_8_O_2_: isotopically labelled butyric acid standard. (C and D) Ion flow diagram and MS analysis of the full MS mode of butyric acid in the heart tissues of ApoE^-/-^ mice at different time periods after the oral administration of ^13^C-labelled butyric acid.


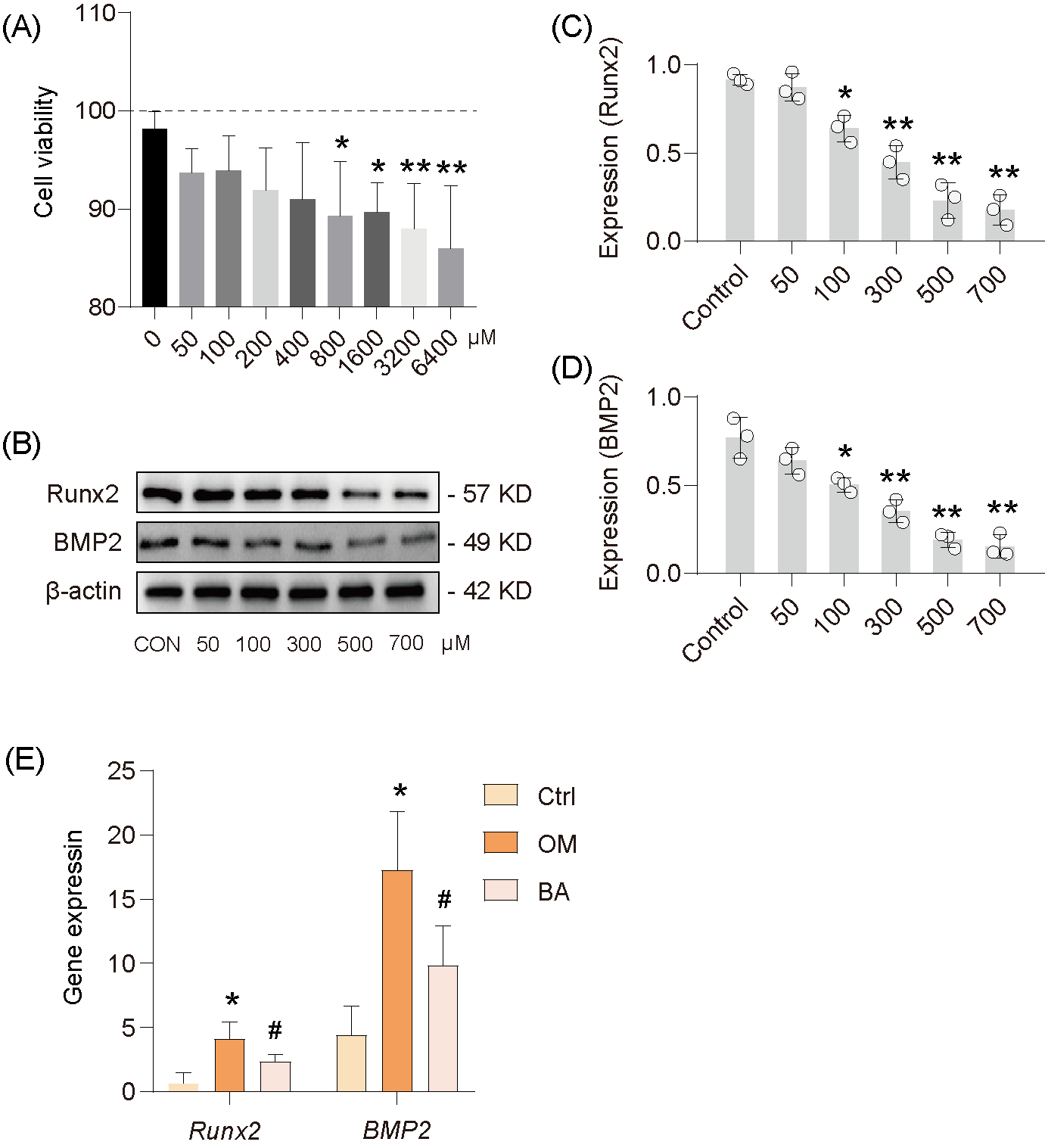


**Figure S8 Effects of butyric acid on cell viability and the mRNA expression levels of *Runx2* and *BMP2*.** (A) Effects of butyric acid on the viability of hVICs. (B-D) Effects of butyric acid on the protein expression levels of Runx2 and BMP2 in hVICs. (E) Gene expression levels of *Runx2* and *BMP2* in hVICs in the control, OM and BA groups. **p* < 0.05, ***p* < 0.01, ****p* < 0.001 indicate significant differences compared with the control group. ^#^*p* < 0.05 indicates significant differences compared with the OM group.


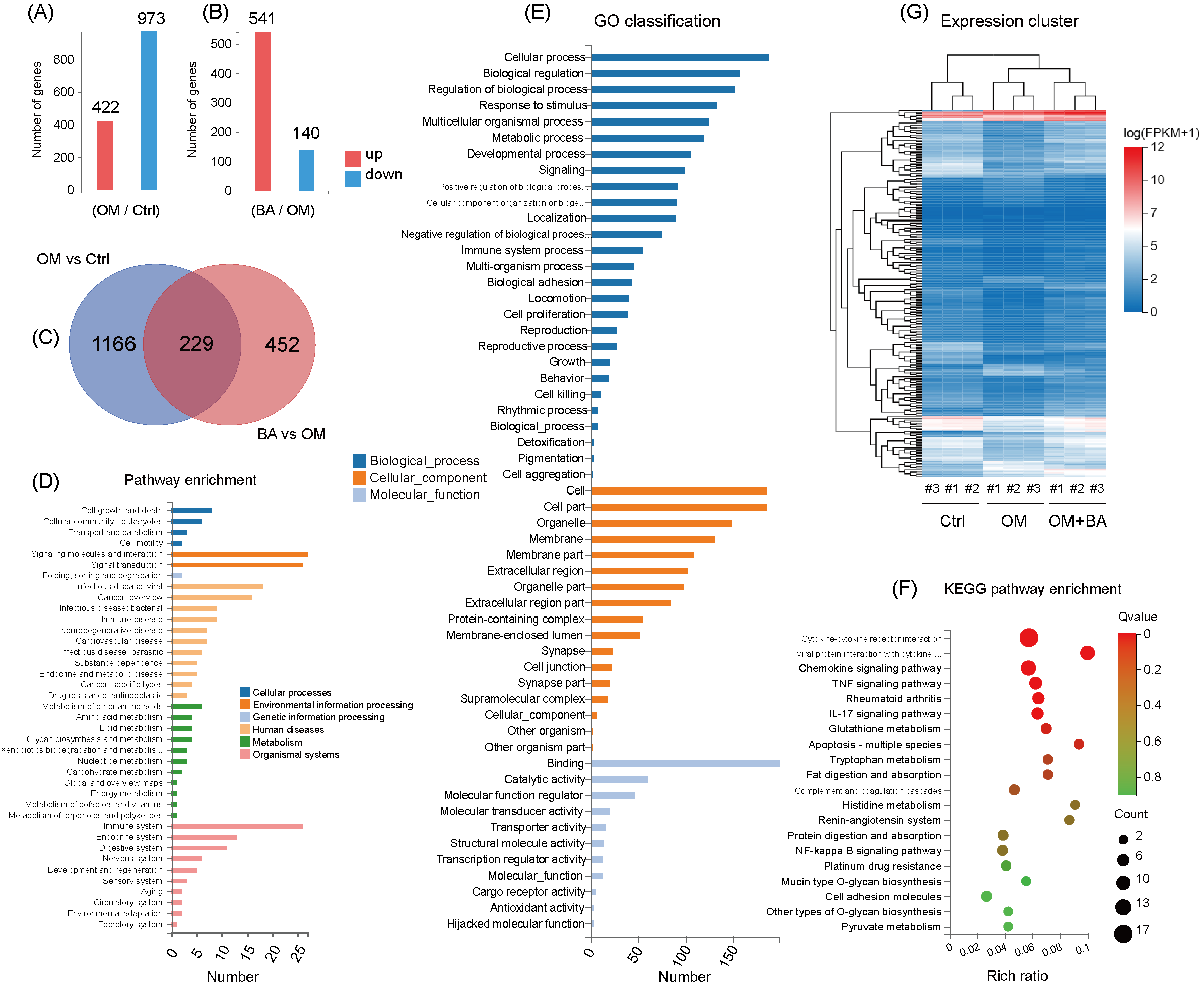


**Figure S9 Gene expression profiles of hVICs in the control, OM and BA groups.** (A) DEGs in hVICs treated with OM compared with the control group. (B) DEGs in hVICs treated with OM + BA (BA group) compared with the OM group. (C) Common DEGs between the DEGs in the OM group compared with the control group and the DEGs in the BA group compared with the OM group. (D) Pathway enrichment analysis of the common DEGs. (E) GO analysis of the common DEGs. (F) KEGG analysis of the common DEGs shown in the gradient diagram. (G) Expression cluster analysis of the common DEGs in the control, OM and OM + BA groups.

**
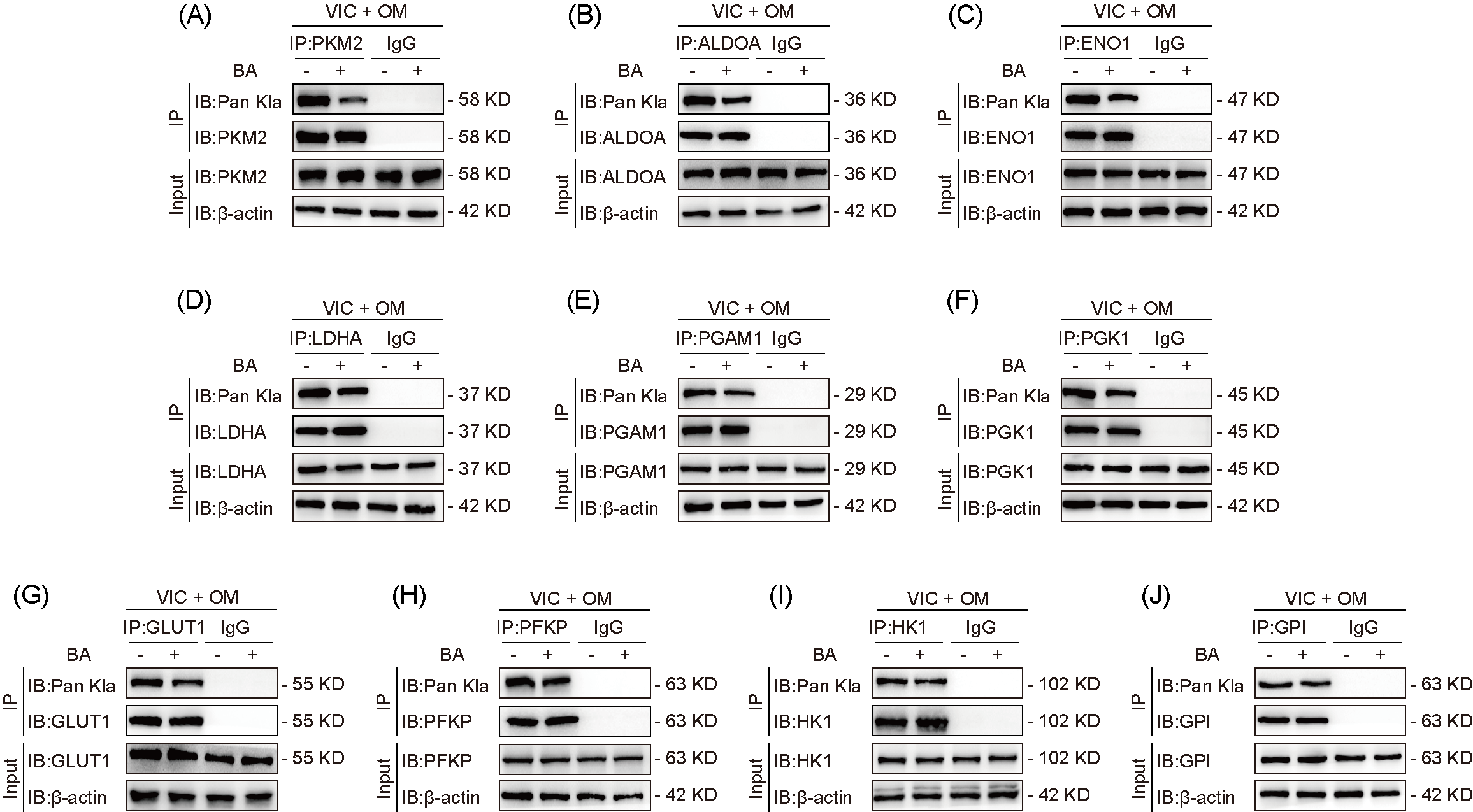
**

**Figure S10 Effect of butyric acid treatment on the level of lactylation of glycolytic enzymes in OM-induced hVICs.** (A) Immunoprecipitation analysis of butyric acid treatment affecting the lactylation level of PKM2, *n* = 3. (B) Immunoprecipitation analysis of butyric acid treatment affecting the lactylation level of ALODA, *n* = 3. (C) Immunoprecipitation analysis of butyric acid treatment affecting the lactylation level of ENO1, *n* = 3. (D) Immunoprecipitation analysis of butyric acid treatment affecting the lactylation level of LDHA, *n* = 3. (E) Immunoprecipitation analysis of butyric acid treatment affecting the lactylation level of PGAM1, *n* = 3. (F) Immunoprecipitation analysis of butyric acid treatment affecting the lactylation level of PGK1, *n* = 3. (G) Immunoprecipitation analysis of butyric acid treatment affecting the lactylation level of GLUT1, *n* = 3. (H) Immunoprecipitation analysis of butyric acid treatment affecting the lactylation level of PFKP, *n* = 3. (I) Immunoprecipitation analysis of butyric acid treatment affecting the lactylation level of HK1, *n* = 3. (J) Immunoprecipitation analysis of butyric acid treatment affecting the lactylation level of GPI, *n* = 3.


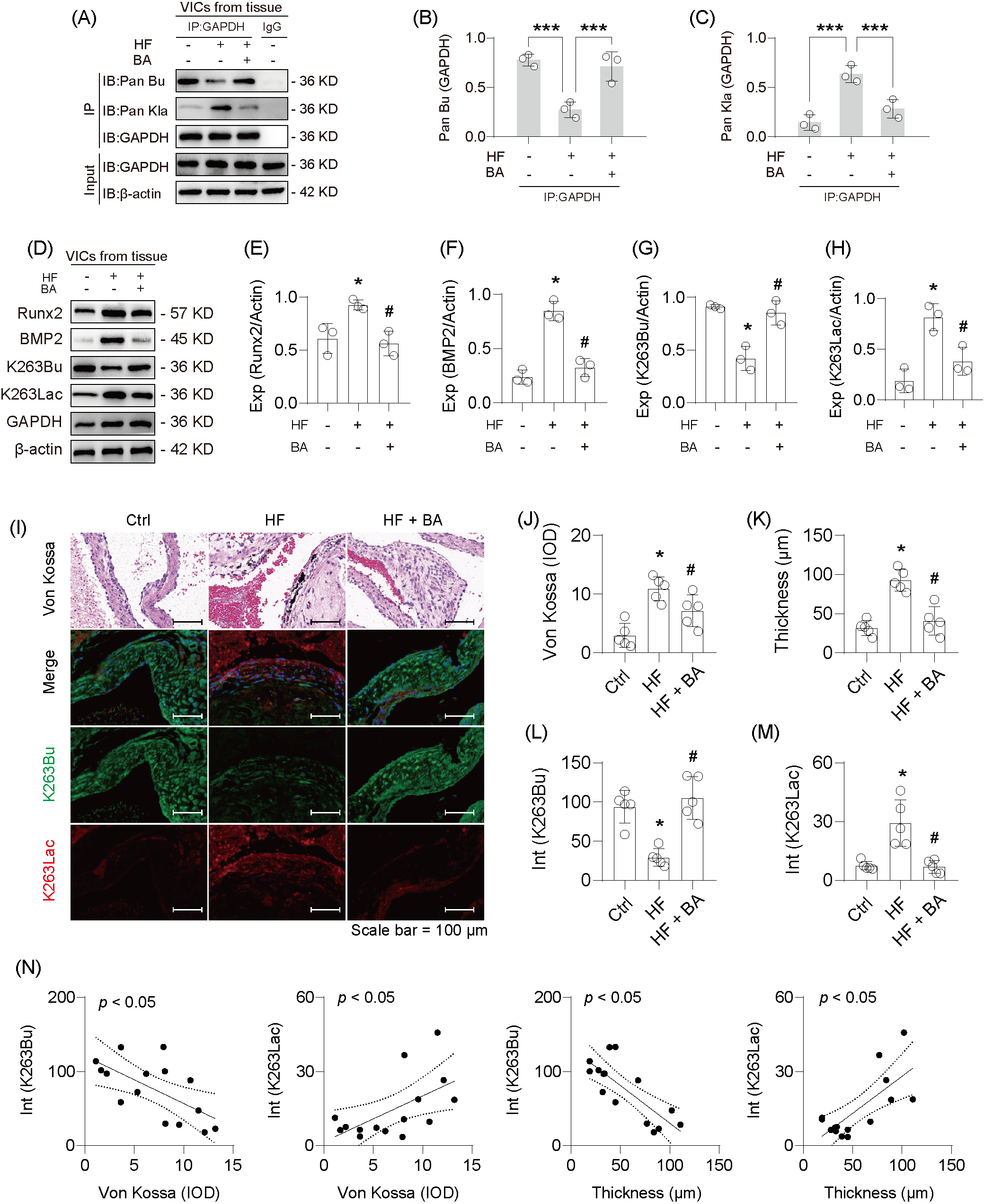


**Figure S11 Butyric acid feeding attenuated valve calcification in high-fat diet-fed mice.** (A-C) Immunoprecipitation was used to detect the effects of butyric acid feeding on GAPDH lactylation and butyrylation in the hVICs of valves from high-fat-fed mice. (D-H) Effects of butyric acid feeding on GAPDH K263 lactylation/butyration and Runx2/BMP2 in hVICs extracted from high-fat-fed mice. (I-N) Von Kossa test and immunofluorescence detection, scale bar: 100 μm. **p* < 0.05, ***p* < 0.01, ****p* < 0.001 indicate significant differences compared with the control group.


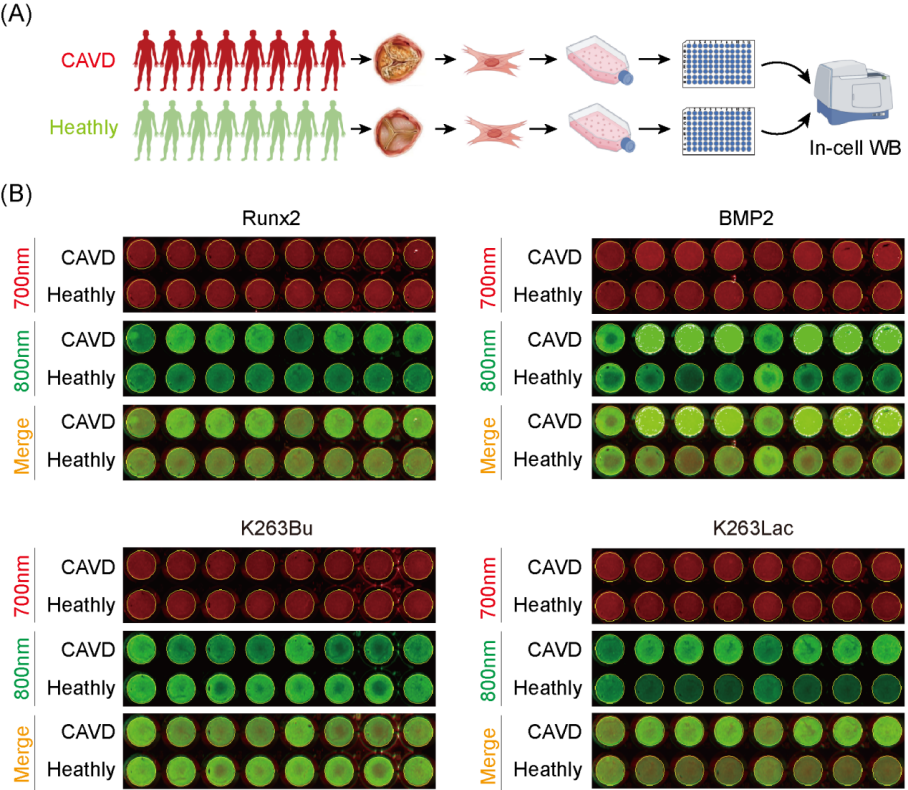


**Figure S12 hVICs were extracted from the valves of 8 calcified patients and 8 healthy patients for in-cell WB detection of the calcification markers Runx2/BMP2 and GAPDH K263 butyrylation/lactylation.** (A) Flow chart of the in-cell WB. (B) The expression of calcification markers Runx2/BMP2 and GAPDH K263 butyrylation/lactylation detcted by in-cell WB.
